# Mycotoxin Contamination: Occurrence, Biotransformation, Pathogenic Mechanisms, and Strategies for Nutritional Intervention

**DOI:** 10.3390/molecules30193860

**Published:** 2025-09-23

**Authors:** Chenyu Yao, Mengyu Ye, Cong Wang, Lin Zou, Ximeng Zhang, Xin Chai, Huijuan Yu, Chengyu Zhang, Yuefei Wang

**Affiliations:** 1 State Key Laboratory of Chinese Medicine Modernization, Tianjin Key Laboratory of TCM Chemistry and Analysis, Tianjin University of Traditional Chinese Medicine, Tianjin 301617, China; yaochenyu0609@163.com (C.Y.); yemengyu2022@163.com (M.Y.); wangcong_1217@126.com (C.W.); linzou230505@126.com (L.Z.); zxmjrpasl@163.com (X.Z.); chaix0622@tjutcm.edu.cn (X.C.); huijuanyu@tjutcm.edu.cn (H.Y.); 2 Haihe Laboratory of Modern Chinese Medicine, Tianjin 301617, China

**Keywords:** mycotoxins, biosynthesis, biotransformation, toxicity, detoxification, nutritional intervention

## Abstract

Mycotoxins, toxic fungal secondary metabolites, exhibit a diverse array of toxicological effects, including hepatotoxicity, carcinogenicity, estrogenicity, immunotoxicity, and neurotoxicity. These toxins cause severe contamination in food, feed, and traditional Chinese medicines (TCMs), threatening global food security and imposing substantial economic burdens. Among over 400 distinct mycotoxins identified to date, aflatoxin B1 (AFB1), ochratoxin A (OTA), and zearalenone (ZEN) stand out for their pervasive contamination and grave toxicities. Upon absorption, these toxins undergo biotransformation into reactive metabolites that exert multifaceted toxicities via mechanisms such as carcinogenesis, estrogenic effects, oxidative stress, inflammation, and abnormal apoptosis, collectively threatening human and livestock health. The application of natural and engineered enterosorbents suppresses intestinal absorption and subsequent bioactivation of mycotoxins, while dietary small-molecule bioactive compounds neutralize post-absorption toxicity via biotransformation intervention and cytoprotective reinforcement, collectively preventing the onset and progression of related diseases. This paper reviews the biosynthetic routes of three representative mycotoxins (AFB1, OTA, and ZEN), along with their biotransformation and underlying pathogenic mechanisms. Furthermore, nutritional intervention approaches targeting the underlying mechanisms to ameliorate mycotoxin-induced damage are discussed. This review not only provides valuable insights for future research on mycotoxin toxicity, but also establishes a theoretical foundation for utilizing dietary strategies to counteract mycotoxin-induced physical damage.

## 1. Introduction

Mycotoxins are toxic secondary metabolites mainly produced by several important fungal genera, including *Aspergillus*, *Penicillium*, and *Fusarium* [1]. Among over 400 distinct types of mycotoxins characterized, aflatoxins (AFs), ochratoxins (OTs), zearalenone (ZEN), deoxynivalenol, fumonisins, and citrinin are considered the most concerning mycotoxins for both human and livestock health (Table S1) [2,3,4,5,6]. Cereals and traditional Chinese medicines (TCMs), easily accessible to the public, are highly susceptible to fungal contamination both prior to harvesting and during subsequent stages of processing, packaging, distribution, and storage [7,8]. Significantly, the Food and Agriculture Organization of the United Nations (FAO) has reported that about 25% of cereals in the world are contaminated by mycotoxins annually [9]. This contamination not only reduces food availability but also contributes to substantial economic losses. Furthermore, it poses a risk of long-term exposure to mycotoxins for both humans and animals, thereby representing an escalating threat to global food security and public health. Mycotoxins exhibit a wide array of toxic effects of on health, such as carcinogenicity, teratogenicity, hepatotoxicity, neurotoxicity, and reproductive toxicity [10]. Given their widespread prevalence and well-documented toxicity, this review specifically focuses on three mycotoxins: aflatoxin B1 (AFB1), ochratoxin A (OTA), and zearalenone (ZEN).

AFs are the most toxic and carcinogenic contaminants produced by fungi such as *Aspergillus flavus* and *Aspergillus parasiticus*, and are commonly found in moldy corn, soybeans, rice, peanuts, and other relevant by-products [11]. Among the more than 20 analogs of AFs, AFB1 is a natural carcinogen with the highest levels of prevalence, toxicity, and carcinogenic potential [12,13]. Consumption of contaminated products may lead to various health risks, including carcinogenicity, teratogenicity, mutagenicity, hepatotoxicity, etc. The International Agency for Research on Cancer (IARC) has classified AFB1 as a Group I carcinogen (a human carcinogen) [14].

OTs are produced by *Aspergillus* or *Penicillium* (mainly *Aspergillus ochraceus* and *Penicillium verrucosum*) under variable environmental conditions, with OTA being the another of the most hazardous and prevalent after AFs [15]. OTA has been known to extensively contaminate numerous food products and beverages, such as cereals, wine, beer, and animal-derived products [16]. Exposure to OTA has been linked to multiple adverse health effects, including nephrotoxicity, hepatotoxicity, mutagenicity, teratogenicity, and immunotoxicity, and IARC has classified it as a Group IIB carcinogen (possible human carcinogen) [17,18].

ZEN, a non-steroidal estrogenic mycotoxin primarily secreted by *Fusarium graminearum* and *Fusarium culmorum*, has raised additional concerns by virtue of its pronounced interaction with estrogen receptors (ERs) [19,20]. Generally, cereals and grain-derived products are susceptible to ZEN contamination, including corn, wheat, rice, barley, and their products. Additionally, milk and dairy products may be contaminated when feedstuff contain high concentrations of ZEN [21]. Numerous studies have confirmed that ZEN exposure demonstrates reproductive toxicity, immunotoxicity, teratogenicity, carcinogenicity, and genotoxicity [22]. It has been classified by IARC as a Group III carcinogen.

Given their substantial health risks and extensive prevalence, it is imperative to grasp the adverse effects and related pathogenic mechanisms of mycotoxins, and to develop effective therapeutic strategies aimed at addressing the associated disease risks. Therefore, through a systematic literature search incorporating keywords such as mycotoxins, biosynthesis, biotransformation, toxicity, detoxification, and nutritional intervention, this review systematically summarizes the biosynthetic and biotransformation pathways of mycotoxins, along with their underlying pathogenic mechanisms. Furthermore, we emphasize the significance of nutritional intervention and elucidate the potential protective mechanisms involved. It is hoped that our review will deepen the comprehension of the harmful impacts and molecular mechanisms of mycotoxins, and strategies for preventing or mitigating diseases associated with mycotoxin exposure in both human and animal populations.

## 2. Biosynthetic and In Vivo Metabolic Pathways

A comprehensive understanding of mycotoxin biosynthesis and their in vivo toxicokinetics—including absorption, distribution, biotransformation, and excretion—is fundamental for developing effective strategies to mitigate mycotoxin contamination and associated health risks. Accordingly, this part systematically reviews the biosynthetic routes of three major mycotoxins in representative fungal strains, and their metabolic fates in mammalian systems, with a focus on humans and livestock.

### 2.1. Biosynthesis and In Vivo Metabolic Pathways of AFB1

AFs are the products of complex biosynthetic pathways involving at least 27 enzymatic reactions. The genes encoding for these enzymes are grouped in a gene cluster, near the telomere of chromosome 3 [23]. This cluster contains not only the genes encoding necessary enzymes for biosynthesis, but also two cluster-specific regulatory genes—*aflR* and *aflS*—that are responsible for transcriptional activation [23,24]. The gene *aflR* encodes a sequence-specific DNA-binding binuclear zinc cluster (Zn(II)2Cys6) protein, AflR, which is required for the transcription activation of most characterized AF pathway genes in both *A. flavus* and *A. parasiticus* [25,26]. In addition, *aflS*, regulated by *aflR*, has been reported to modulate early pathway genes and function as a co-activator alongside *aflR* [27,28]. The detailed information regarding both the old and new nomenclatures of cluster genes, along with their functional roles across various representative fungal species, is presented in Table S2.

**Figure 1:**
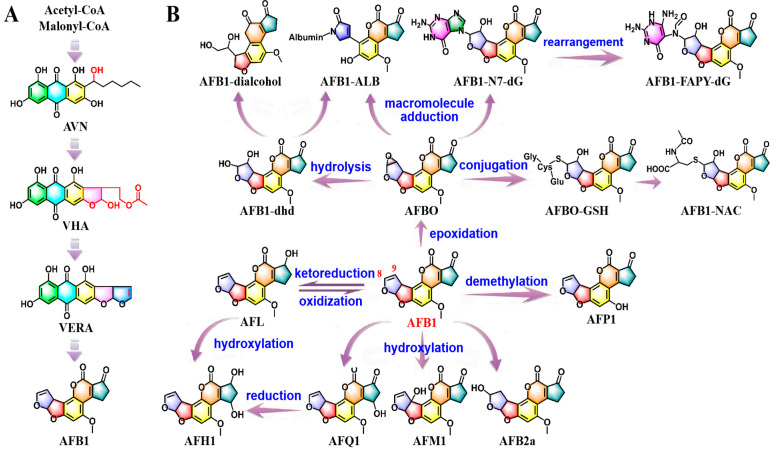
Schematization of AFB1 biosynthetic route with main intermediate metabolites and associated functional genes (**A**); bioactivation and detoxification pathways of AFB1 (**B**).

AFB1 biosynthesis begins with the stepwise conversion of hexanoate units into norsolorinic acid anthrone (NAA), norsolorinic acid (NOR), and averantin (AVN) by proteins encoded by five genes: *aflA*, *aflB*, *aflC*, *hypC*, and *aflD* [28]. Subsequently, *aflG*, *aflH*, and *aflK* sequentially participate in the transformation of AVN into 5′-hydroxyl-averantin (HAVN), 5′-oxoaverantin (OAVN), and averufin (AVF) [29,30]. The conversion of AVF to hydroxyversicolorone (HVN) and versiconal hemiacetal acetate (VHA) is catalyzed by *aflV* and *aflW* [31]. Interestingly, the isolation and characterization of *aflI* have revealed that this gene is capable of direct conversion of AVF into VHA [28]. *aflJ* encodes an esterase catalyzing the reaction converting VHA to versiconal (VAL), which has also been reported to allow the reversible transformation of VHA into versiconol acetate (VOAc) and from versiconol (VOH) to VAL [23]. The sequential transformation of VAL into versicolorin B (VERB) and versicolorin A (VERA) are mediated by the products of *aflK* and *aflL* [30,32]. *aflM*, *aflN*, *aflY*, and *aflX* are predicted to convert VERA into demethylsterigmatocystin (DMST) [33,34,35]. Furthermore, *O*-methyltransferase B methylates DMST to produce sterigmatocystin (ST), which is then converted to *O*-methylsterigmatocystin (OMST) by *O*-methyltransferase A [29,36,37]. The final conversion of OMST to AFB1 is catalyzed by the following genes: *aflQ*, *hypD*, *hypB*, *aflE*, and *hypE* (Figure 1 and Figure S1).

As a pro-carcinogen, the hepatic biotransformation of AFB1 is essential for its toxic and carcinogenic effects, proceeding through two phases: phase-I metabolic activation and phase-II detoxification reactions. Phase-I metabolic activation, primarily mediated by cytochrome P450 (CYP450) enzymes (including CYP1A1, CYP1A2, CYP2A5, CYP2A6, CYP2A13, CYP2E1, CYP3A4, CYP3A5, CYP3A7, CYP3A11, and CYP3A13), generates reactive intermediates that disrupt cellular homeostasis and drive toxicity through multiple mechanisms [38,39]. Furthermore, the nuclear receptor superfamily (NR) is implicated in the regulation of CYP450 expression. Specially, aryl hydrocarbon receptor (AHR), constitutive androstane receptor (CAR), and pregnane X receptor (PXR) are responsible for modulating the expression of CYP1, CYP2, and CYP3 subfamilies, respectively [40]. Conversely, phase-II biotransformation reactions are often recognized as detoxification and elimination pathways. These processes involve the conjugation of AFB1 and its metabolic derivatives with nucleophilic molecules, such as reduced glutathione (GSH), reactions catalyzed by glutathione-S-transferases (GSTs). Among the various GSTs, GSTA1, GSTA2, GSTA3, GSTA4, GSTA5, GSTM1, GSTM2, and GSTT1 are important members to neutralize AFB1 toxicity by enhancing its metabolic clearance. Additionally, microsomal epoxide hydrolase (mEH) and aflatoxin–aldehyde reductase (AFAR) serve as crucial phase-II enzymes in AFB1 detoxification [38]. The toxicity level of AFB1 is influenced by both the rates of formation and elimination of these harmful metabolites.

Significantly, the epoxidation of AFB1 leads to the formation of the highly reactive and electrophilic compound AFB1-exo-8, 9-epoxide (AFBO), which appears to initiate the pronounced toxicity and carcinogenicity of AFB1. Furthermore, AFB1 undergoes biotransformation into moderately toxic aflatoxicol (AFL), mildly toxic aflatoxin M1 (AFM1), and relatively non-toxic aflatoxin P1 (AFP1) or aflatoxin Q1 (AFQ1) [38]. Intriguingly, AFL can be enzymatically reconverted back to AFB1, acting as a reservoir of AFB1 to prolong its toxic effects [41]. Limited information is available regarding the production of aflatoxicol H1 (AFH1), but it is likely formed from a reduction of AFQ1 or a hydroxylation of AFL [42]. Moreover, symbiotic microbes in the gut or rumen have also been reported to participate in the transformation of aflatoxin B2a (AFB2a) [43] (Figure 1).

Once formed from AFB1, AFBO undergoes three major routes during phase-II reactions: GSH conjugation, macromolecule adduction, and hydrolysis. AFBO can be metabolically detoxified through conjugation with GSH, leading to the formation of AFBO-GSH. Subsequently, through the action of γ-glutamyltranspeptidase (GGT), dipeptidase (DPEP), and N-acetyltransferase (NAT), AFBO-GSH is further metabolized to the water-soluble non-toxic AFB1-mercapturic acid (AFB1-NAC), which is ultimately excreted in the urine [41,42]. This reactive epoxide intercalates into the DNA double helix and covalently bonds with the most nucleophilic N7 atom of deoxyguanosine (dG) to form an AFB1-N7-dG adduct [13]. Due to the positive charge on the imidazole ring, the DNA adduct is chemically unstable and can further rearrange into the open ring form of AFB1-formamidopyrimidine-dG (AFB1-FAPY-dG) adduct or depurination to generate an abasic site (AP) [13,44]. Part of AFBO covalently binds to the ε-amino group of lysine residues in serum albumin, resulting in the formation of AFB1-albumin (AFB1-ALB) adduct [45]. In addition to GSH conjugation and macromolecular adduction, AFBO can be hydrolyzed through either spontaneous processes or enzymatic catalysis by mEH, producing the less toxic metabolite AFB1-8,9-dihydrodiol (AFB1-dhd) [34]. This compound still retains its ability to bind to lysine residues [41]. Then, AFB1-dhd can be metabolized into real detoxified product AFB1-dialcohol by AFAR (Figure 1).

### 2.2. Biosynthesis and In Vivo Metabolic Pathways of OTA

Unlike other significant mycotoxins, the biosynthetic pathway of OTA has not been fully and comprehensively elucidated. While most molecular aspects of OTA biosynthesis have been clarified in *Penicillium*, they have only been partially addressed in *Aspergillus* species [46,47]. Wang et al. [48] characterized the OTA biosynthetic gene cluster of *A. ochraceus* fc-1. This gene cluster contains five highly conserved genes, including *otaA*, *otaB*, *otaC*, *otaD*, and *otaR1*. In addition to these core genes, adjacent sequences also include those encoding a flavin adenine dinucleotide (FAD)-dependent oxidoreductase (*otaE*) and a GAL4-like Zn2Cys6 binuclear DNA-binding protein (*otaR2*). The pathway-specific regulator *otaR1* is involved in the regulation of *otaA*, *otaB*, *otaC*, and *otaD*, while it does not regulate either *otaR2* or *otaE*. The second regulator *otaR2*, specifically modulates only the expression of *otaA*, *otaC*, and *otaD*. The identification of the polyketide synthase (PKS) cyclase gene *otaY*, located between *otaA* and *otaB*, which encodes a SnoaL-like protein, suggests its putative role in the cyclization of polyketide backbones [49]. Comparative literature analysis indicates that the cluster in *A. ochraceus* exhibits similarities to that of *P. verrucosum*, but the latter lacks *otaR2* and *otaY* [50] (Table S2).

**Figure 2:**
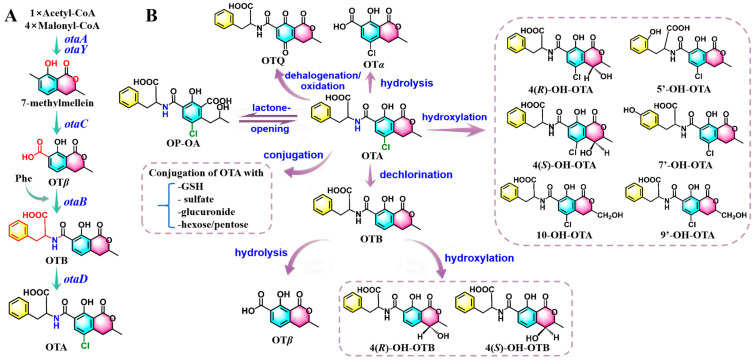
Putative OTA biosynthetic pathway, delineating intermediates and biosynthetic genes (**A**); overview of OTA biotransformation in humans and animals (**B**).

OTA biosynthesis is initiated by the enzymes encoded by *otaA* and *otaY*, utilizing the conversion of acetyl-CoA and malonyl-CoA to 7-methylmellein, which is subsequently oxidized to ochratoxin *β* (OT*β*) through the action of *otaC* [28]. Following this oxidation step, nonribosomal peptide synthase (NRPS) catalyzes the formation of an amide bond between OT*β* and L-*β*-phenylalanine (Phe), marking a crucial stage in the biosynthesis of OTB [48]. The halogenase encoded by *otaD* introduces a chlorine atom at the C-5 position within the final molecular structure of OTA [51] (Figure 2).

OTA has been reported to be metabolized in the kidney, liver, and intestine, but it is preferentially distributed to the kidneys [52]. OTA undergoes biotransformation via both phase-I and phase-II enzymes, with hydrolysis, lactone-opening, hydroxylation, and conjugation identified as key metabolic pathways [52,53]. It is noteworthy that the phase-I reactions are generally associated with the action of the CYP450 enzyme family, involving CYP1A1, CYP1A2, CYP1A5, CYP2B1, CYP2D6, CYP2C18, CYP3A1, CYP3A2, CYP3A4, and CYP3A9 isoforms [40,52,53,54].

In the intestine, OTA can be hydrolyzed to non-toxic Ochratoxin *α* (OT*α*) through the catalysis of proteolytic enzymes and enzymes from the bacterial microflora [52,53]. Under alkaline conditions, hydrolysis may provoke reversible conformational changes in the lactone ring, which results in the formation of the resultant toxic end product lactone-opened OTA (OP-OA) [55]. Additionally, hydroxylation serves as a significant metabolic pathway that generates various less toxic derivatives, including 4(*R*)-OH-OTA, 4(*S*)-OH-OTA, 5′-OH-OTA, 7′-OH-OTA, 9′-OH-OTA, and 10-OH-OTA [52,56]. Dechlorination of OTA leads to the formation of OTB with a comparatively less genotoxic effect, which can be subsequently converted into OT*β*, 4(*R*)-OH-OTB, and 4(*S*)-OH-OTB [52,54,57]. Oxidation of OTA by CYP450 enzymes generates a reactive electrophilic product, OTA-quinone (OTQ), which may undergo partial detoxification via conjugation with GSH or reduction to form OTA-hydroquinone (OTHQ) [58]. In phase-II reactions, detoxification enzymes facilitate the formation of OTA-conjugated complexes, playing critical roles in the elimination processes. Such complexes primarily include GSH, sulfate, glucuronide, and hexose/pentose conjugations of OTA [52,53] (Figure 2).

### 2.3. Biosynthesis and In Vivo Metabolic Pathways of ZEN

Until now, only four genes have been molecularly characterized as functional contributors to the biosynthetic pathway of ZEN, including *PKS4*, *PKS13*, *ZEB1*, and *ZEB2* [28,59]. Specifically, *PKS4*, *PKS13*, and *ZEB1* encode distinct catalytic components essential for the biosynthetic machinery: reducing PKS, non-reducing PKS, and isoamyl alcohol oxidase, respectively [60,61]. However, *ZEB2* encodes a basic leucine zipper (bZIP) transcription factor that is responsible for the regulation of the other three genes [28]. These neighboring genes form a cluster, coordinately regulated at the transcriptional level [60] (Table S2).

**Figure 3:**
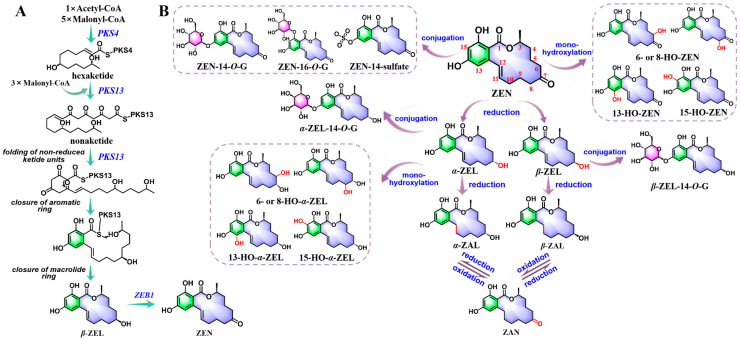
Proposed pathways for the biosynthesis of ZEN (**A**); schematization of ZEN metabolites during phase-I and phase-II processes (**B**). Given that the knowledge regarding the formation of dihydroxylated ZEN and O-ZEN remains ambiguous, we have refrained from labeling the relevant information on the transformation diagram.

During ZEN biosynthesis, *PKS4* mediates the condensation of carbons from a single acetyl-CoA molecule and five malonyl-CoA molecules to produce a hexaketide [59]. *PKS13* catalyzes the extension of the ZEN chain by incorporating three extra malonyl-CoA molecules resulting in a nonaketide, and facilitates the folding of non-reduced ketide units [20,28,62]. Subsequently, the unreduced ketones undergo two series of intramolecular aromatic reactions, leading to the formation of an aromatic ring and a macrolide ring structure with a lactone bond [59,62]. Ultimately, *β*-zearalenol (*β*-ZEL) is oxidized to ZEN with assistance by *ZEB1* (Figure 3).

The liver and intestine constitute the primary sites responsible for ZEN metabolic processing [63]. Initially, under the catalysis of 3*α*-/3*β*-hydroxysteroid dehydrogenases (HSDs), ZEN undergoes reduction reactions at the C7-keto group, leading to the formation of *α*-zearalenol (*α*-ZEL) and *β*-ZEL. The following saturation of the C11-C12 conjugated double bonds then generates *α*-zearalanol (*α*-ZAL) and *β*-zearalanol (*β*-ZAL), which are capable of engaging in reversible redox conversion to zearalanone (ZAN) [63,64]. Furthermore, several oxidized metabolites of ZEN and its reduced derivatives have been detected, including monohydroxylated and dihydroxylated derivatives, along with a potentially novel metabolite O-ZEN [65]. Among these, the monohydroxylated derivatives have been more extensively characterized. Possible monohydroxylated metabolites of ZEN and *α*-ZEL were demonstrated [66], which confirmed aliphatic hydroxylation products (6- or 8-HO-ZEN and 6- or 8-HO-*α*-ZEL) and aromatic hydroxylation derivatives (13-HO-ZEN, 15-HO-ZEN, 13-HO-*α*-ZEL, and 15-HO-*α*-ZEL). Furthermore, a dihydroxylated derivative has been discovered, although its hydroxylation sites and structural configuration remain ambiguous [65]. Additionally, the formation mechanism of O-ZEN remains to be elucidated. A plausible biosynthetic route involves catecholic intermediates, such as 13-HO-ZEN or 15-HO-ZEN, which subsequently undergo non-enzymatic autoxidation to the quinone form (Figure 3).

Glucuronidation and sulfation have emerged as the predominant phase-II metabolic pathways for ZEN and its derivatives. Such processes are mediated through enzymatic conjugation with glucuronic acid via UDP glucuronosyltransferases (UGT) and sulfate by sulfotransferases (SULTs) [65]. Key isoforms involved include UGT1A1, UGT1A3, UGT1A8, UGT2B7, SULT2A1, and SULT2B1 [65,67]. The principal conjugates include ZEN-14-*O*-glucuronide (ZEN-14-*O*-G), ZEN-16-*O*-G, ZEN-14-sulfate, *α*-ZEL-14-*O*-G, *β*-ZEL-14-*O*-G, and so on [68] (Figure 3).

## 3. Mechanisms of Mycotoxin Pathogenesis

A comprehensive elucidation of mycotoxin pathogenesis at the molecular level is indispensable for establishing a theoretical foundation for mitigating toxin-induced physiological damage and advancing the development of effective nutritional intervention against these toxins.

### 3.1. Structure-Related Toxicopathological Mechanisms of Mycotoxins

Specific structural features of mycotoxins may mediate their toxicological outcomes: the epoxidation of the unsaturated double bond at the C8-C9 position of AFB1 contributes to its hepatocarcinogenic potential; the Phe moiety and isocoumarin moiety in OTA facilitate protein synthesis inhibition; and the structural similarity of ZEN to 17*β*-estradiol enables its estrogenic effects.

#### 3.1.1. AFB1-Induced Hepatocarcinogenesis by Genotoxic Adduct Formation, Oxidative Mutagenesis, and Epigenetic Dysregulation

**Figure 4:**
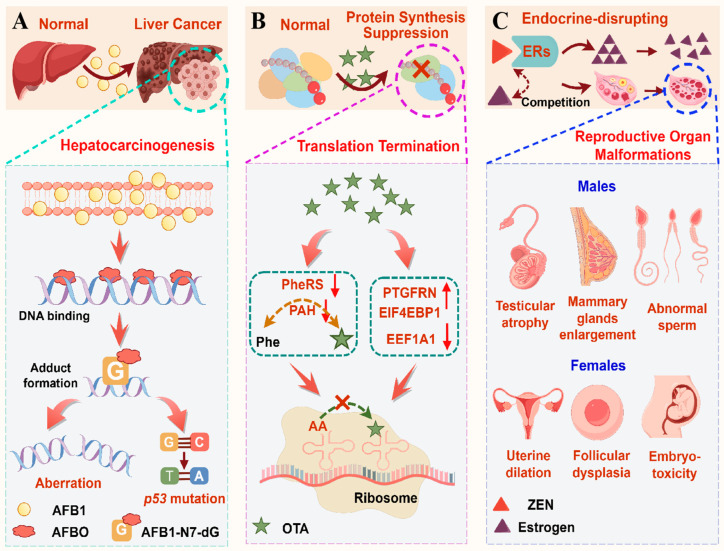
Structure-related toxicopathological mechanisms of AFB1, OTA, and ZEN. AFB1-driven hepatocarcinogenesis: AFB1 is metabolized into the reactive electrophile AFBO, which forms AFB1-N7-dG adducts by binding to the N7 atom of dG. These adducts impair the structural integrity of DNA and hinder both DNA replication and repair processes, leading to mutations such as a G-to-T mutation at p53 codon 249 (**A**); OTA-induced protein synthesis suppression: OTA inhibits PheRS and PAH via its Phe and isocoumarin moieties, leading to abnormal Phe accumulation and disrupted amino acid catabolism. It also upregulates PTGFRN and EIF4EBP1, while downregulating EEF1A1. This modulation disrupts the GTP-dependent binding of aminoacyl-tRNA to the ribosomal A-site, ultimately suppressing global protein synthesis (**B**); ZEN-mediated estrogenic disruption: due to structural similarity with 17*β*-estradiol, ZEN and its metabolites competitively bind to ERs, ultimately activating EREs and disrupting endogenous estrogen homeostasis. This leads to reproductive pathologies: testicular atrophy, enlargement of the mammary glands, and abnormal semen in males; and uterine dilation, follicular dysplasia, and embryotoxicity in females (**C**).

The epoxidation of the unsaturated double bond at the C8-C9 position is the most critical metabolic reaction that contributes to carcinogenic and mutagenic effects [69]. The resultant AFB1-N7-dG adducts compromise the structural integrity of DNA and hinder both DNA replication and repair processes, leading to point mutations, insertion–deletion events, or chromosomal aberrations [44]. Furthermore, mutagenesis arising from the replication of a damaged DNA template within tumor suppressor genes or oncogenes is closely associated with the onset of cancer [44]. AFB1-FAPY-dG and AP may result in genetic mutations, with the most prevalent being a G-to-T mutation at the third position in codon 249 (AGG) of the *p53* tumor suppressor gene [42,69]. Concurrently, the cytotoxicity of AFB1-ALB protein binding further contributes to tissue damage, resulting in a pro-inflammatory and pro-proliferative state, thereby promoting cancer [42]. 8-hydroxydeoxyguanosine (8-OHdG), generated from the reaction of hydroxyl radicals with guanine residues in DNA, is a mutagenic DNA oxidative damage product and plays an important role in the chemical carcinogenesis [69]. Epigenetically, AFB1 suppresses the activity of histone acetyl transferase (HAT) while simultaneously activating histone deacetylase (HDAC), leading to histone hypoacetylation and chromatin compaction to consequently silence certain tumor suppressor genes and DNA repair genes [70] (Figure 4A).

#### 3.1.2. OTA-Induced Protein Synthesis Suppression by Phe/Isocoumarin-Mediated Dual Enzyme Inhibition Coupled with Ribosomal Translational Arrest

OTA is structurally characterized by a Phe moiety and an isocoumarin moiety interconnected via an amide bond [18]. Studies have demonstrated its inhibitory effect on phenylalanine tRNA synthase (PheRS) activity. While early hypotheses proposed that the Phe moiety of OTA acts as a competitor against Phe for binding, subsequent studies indicated that the isocoumarin moiety plays a more critical role in the interaction [53]. Additionally, OTA functions as an inhibitor of phenylalanine hydroxylase (PAH), with its Phe moiety acting as a false substrate of the catalytic reaction [71]. This interference disrupts Phe-to-tyrosine hydroxylation, leading to abnormal Phe accumulation and impaired amino acid catabolism. OTA upregulates prostaglandin F2 receptor negative regulator (PTGFRN) and eukaryotic translation initiation factor 4E binding protein-1 (EIF4EBP1), while downregulating elongation factor 1-alpha 1 (EEF1A1). This modulation disrupts the guanosine triphosphate (GTP)-dependent binding of aminoacyl-tRNA to the ribosomal A-site, ultimately suppressing global protein synthesis [72] (Figure 4B).

#### 3.1.3. ZEN-Mediated Estrogenic Disruption by Endocrine Homeostasis Impairment and Pathological Alterations

The structural homology with 17*β*-estradiol enables ZEN and its metabolites to competitively bind to ERs, thereby activating estrogen response elements (EREs). This interaction disrupts endogenous estrogen homeostasis and ultimately drives pathological alterations in reproductive tissues [73]. ZEN exposure decreases the levels of testosterone, progesterone, luteinizing hormone, and follicle-stimulating hormone [21,74]. Furthermore, ZEN induces feminization of testicular atrophy and enlargement of the mammary glands in males [64]. It impairs spermatogenesis, decreases semen quality, and declines sperm concentration, motility, acrosome reaction, and viability while increasing sperm malformation and mortality [74]. Collectively, these cumulative deficits may contribute to diminished fertilization potential and compromised fertility. In parallel, the estrogenic effects of ZEN in females include fertility disorders, ovarian and uterine dilation, vaginal prolapse, vulvar swelling, and breast enlargement [21,64]. It also compromises ovarian development by depressing follicular maturation, inducing follicular atresia, and disrupting oocyte meiosis [75,76]. Additionally, exposure to ZEN during pregnancy may adversely effect offspring development, leading to embryonic malformations and reproductive dysfunction [77] (Figure 4C).

### 3.2. Oxidative Stress

**Figure 5:**
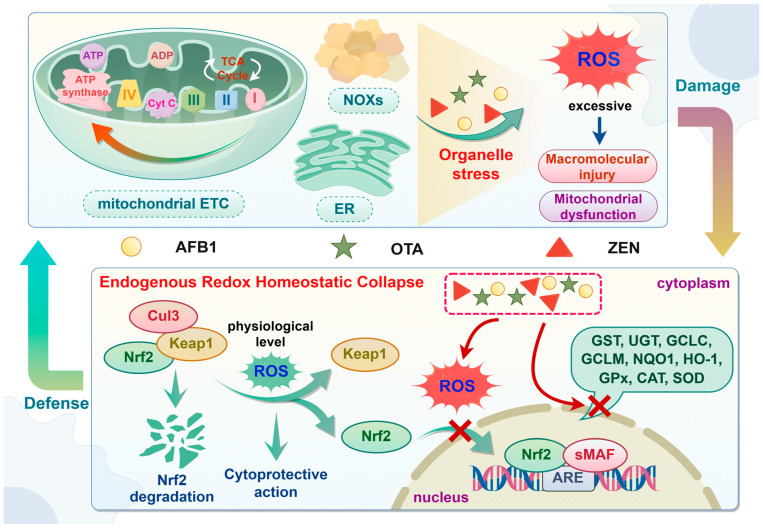
Integrated oxidative stress network of mycotoxins: ROS overproduction coupled with Keap1-Nrf2 signaling suppression. AFB1, OTA, and ZEN trigger ROS overproduction via mitochondrial ETC, ER, and NOX pathways. When ROS generation exceeds homeostatic thresholds, they disrupt redox balance, causing damage to biological macromolecules and mitochondrial function. Additionally, these mycotoxins suppress the activation of Keap1-Nrf2 signaling pathway and block the expression of downstream cytoprotective genes, ultimately impairing endogenous antioxidant detoxification pathways.

Oxidative stress represents an imbalance between oxidants and antioxidants, primarily driven by the excessive production of reactive oxygen species (ROS) and the impairment of antioxidant detoxification pathways [78]. ROS are predominantly generated through mechanisms such as the mitochondrial electron transport chain (ETC), the endoplasmic reticulum (ER), and NADPH oxidases (NOXs) [79]. Physiological levels of ROS play significant roles in signal transduction and transcriptional activation. Nevertheless, when ROS generation exceeds homeostatic thresholds, they disrupt redox balance, causing damage to biological macromolecules, such as phospholipid layer, proteins, and nucleic acid, which impairs their function [78,80]. Significantly, in addition to macromolecular damages, oxidative stress triggers mitochondrial dysfunction, resulting in mitochondrial DNA (mtDNA) mutations, reduced mitochondrial membrane potential (MMP), diminished oxidative phosphorylation capacity, persisting opening of the mitochondrial permeability transition pore (mPTP), and elevated ROS production [78,81,82]. Such processes establish a self-perpetuating cycle wherein oxidative stress amplifies mitochondrial damage, which in turn drives further ROS overproduction and progressive cellular deterioration (Figure 5).

The Kelch-like ECH-associated protein 1-nuclear factor-E2-related factor 2 (Keap1-Nrf2) signaling pathway functions as a master regulatory system that coordinates antioxidant responses to ameliorate redox imbalance. Under homeostatic conditions, Nrf2 is sequestered in cytoplasm by its repressor Keap1, which forms a ubiquitin E3 ligase complex with Cullin 3 (Cul3). This binding promotes Nrf2 ubiquitination and subsequent proteasomal degradation [83,84]. Under oxidative or electrophilic stress, the reactive cysteine residues of Keap1 are modified, which reduces ubiquitin E3 ligase activity and results in Nrf2 stabilization [83,85]. This allows Nrf2 to translocate into the nucleus, heterodimerize with small musculo-aponeurotic fibrosarcoma (sMAF) proteins, bind to antioxidant-responsive elements (AREs), and ultimately initiate the transcription of downstream cytoprotective genes [83]. These include genes encoding phase-II enzymes and endogenous antioxidants, such as those involving *GST*, *UGT*, glutamate-cysteine ligase (*GCL*, including two subunits *GCLC* and *GCLM*), NAD(P)H oxidoreductase 1 (*NQO1*), heme oxygenase-1 (*HO-1*), glutathione peroxidase (*GPx*), catalase (*CAT*), and superoxide dismutase (*SOD*) [84] (Figure 5).

AFB1 triggers oxidative stress primarily via mitochondrial complex-related gene dysregulation, driving intracellular ROS overproduction, while concurrently elevating malondialdehyde (MDA) and depleting GSH, leading to lipid peroxidation and antioxidant imbalance [86]. It suppresses antioxidant defenses by reducing SOD3 content and decreasing activities of GPx, CAT, and total SOD (T-SOD) [87,88]. The Kyoto Encyclopedia of Genes and Genomes (KEGG) analysis shows that AFB1 inhibits the expression of *gsto1*, *gpx4a*, microsomal GST (*mgst3a*), and isocitrate dehydrogenase 1 (*idh1*) in the GSH metabolizing enzyme gene pathway, resulting in hepatic oxidative stress [89]. Additionally, a disturbance in the Keap1-Nrf2 signaling pathway has also been observed in mice kidneys in response to AFB1, as manifested by the downregulation of mRNA expression of genes for enzymatic antioxidant system (*Nrf2*, *CAT*, and *SOD1*) and detoxifying enzymes (*NQO1*, *GCLM*, and *GCLC*), along with increased *Keap1* expression [90].

OTA activates the expression and nuclear translocation of AHR and PXR, subsequently inducing CYP1A1, CYP1A2, and CYP3A4 to exacerbate ROS overproduction through the reaction of CYP450 enzymes with OTA [91]. Concurrently, OTA stimulates mitochondrial-dependent ROS accumulation via the overexpression of hsa-miR-3065-5p, hsa-miR-520g-3p, and hsa-miR-5698, disrupting CACNA1D-mediated p38 mitogen-activated protein kinase (p38 MAPK) signaling pathway [92]. Furthermore, this pro-oxidant state can be exacerbated by suppressed antioxidant defenses, evidenced by decreased activities of SOD, CAT, and GPx, along with depleted GSH and elevated MDA levels [91,93]. OTA exposure upregulates *Keap1* while decreasing the expression of *Nrf2* and its downstream targets, such as *NQO1*, *HO-1*, and *GPx*, thereby impairing cellular redox homeostasis [94]. Paradoxically, conflicting reports suggest that OTA may activate Nrf2 expression and translocation, primarily induced directly by OTA-activated AHR and PXR, or indirectly by adaptive response to ROS generated through metabolic processes [91]. These dual regulatory mechanisms highlight the complexity of the oxidative toxicity of OTA.

ZEN exposure induces mitochondrial dysfunction, characterized by aberrant mitochondrial distribution, impaired MMP, and dysregulated mitochondria-related genes, which may trigger ROS overproduction [76]. Furthermore, it amplifies intracellular ROS through MAPK pathway activation and intracellular calcium ion overload via membrane channel modulation, synergistically exacerbating oxidative damage [73]. Simultaneously, a significant increase in oxidative stress indices (MDA content) and reduction in antioxidant systems (SOD, CAT, GPx, GST activities and GSH level) have also been observed, which further confirm the disturbance of redox homeostasis and the impairment of antioxidant defenses [95]. Additionally, it suppresses Nrf2-mediated antioxidant response by upregulating Keap1a expression, while decreasing the expression of nuclear Nrf2 protein and antioxidant enzymes, such as CAT, HO-1, NQO1, GPx isoforms (GPx1a, GPx1b, GPx4a, and GPx4b), and GST isoforms (GSTR, GSTO1, and GSTO2) [96,97].

### 3.3. Inflammation

Inflammation is a tightly regulated physiological process essential for pathogen clearance and tissue repair, yet its dysregulation drives tissue injury and organ failure [98]. These dual processes can be mediated through multiple signaling pathways, such as MAPK and nuclear factor kappa-B (NF-κB).

**Figure 6:**
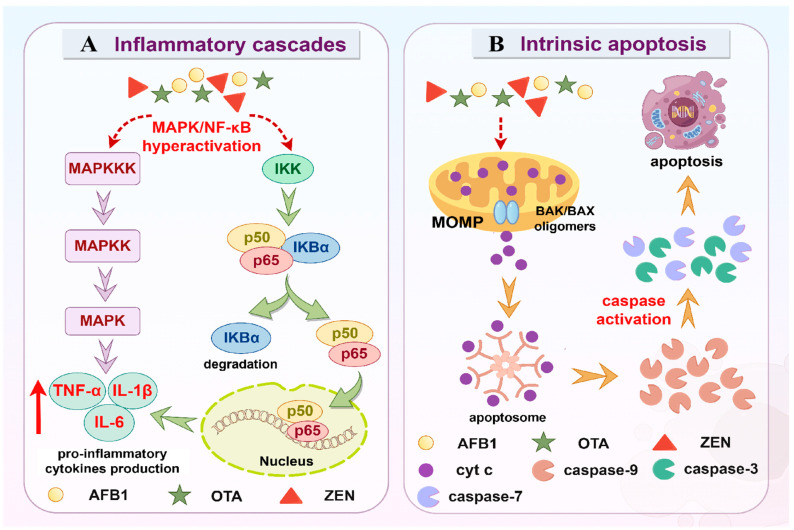
Mycotoxin-triggered inflammatory cascades and aberrant apoptosis. Hyperactivation of MAPK and NF-κB signaling pathways: AFB1, OTA, and ZEN induce excessive activation of the MAPK and NF-κB signaling pathways, thereby promoting the secretion of pro-inflammatory cytokines and exacerbating systemic inflammatory responses. (**A**); dysregulation of intrinsic apoptosis: AFB1, OTA, and ZEN lead to intrinsic apoptotic pathway. (**B**).

MAPK cascades are evolutionarily conserved and play significant roles in activating innate immunity to defend against various pathogens [99]. The conventional MAPK group consists of extracellular signal-regulated protein kinase 1/2/5 (ERK 1/2/5), c-Jun amino (N)-terminal kinase 1-3 (JNK 1/2/3), and p38 MAPKs [100]. Conventional MAPKs form a three-level transduction cascade, specifically, MAPK kinase kinase (MAPKKK) directly phosphorylates and activates MAPK kinase (MAPKK), which in turn activates MAPK by dual phosphorylation of the conserved threonine-X-tyrosine (Thr-X-Tyr) motif. Once activated, MAPK phosphorylates diverse substrates to modify gene expression and protein function that execute appropriate biological responses [101]. Moreover, the ubiquitous transcription factor NF-κB functions as a pivotal mediator of inflammatory responses [102]. Regarding the canonical NF-κB pathway, in the resting state, NF-κB p65 and NF-κB p50 are sequestered in the cytoplasm by the IκB (inhibitor of NF-κB) proteins [103]. Upon activation, IκB kinase (IKK) complex phosphorylates IκBα and triggers its ubiquitin-dependent degradation by proteasomes, liberating NF-κB p65 and NF-κB p50 to translocate to the nucleus and consequently activate the downstream target genes [102,103]. Hyperactivation of MAPK and NF-κB signaling leads to excessive pro-inflammatory cytokine production, such as tumor necrosis factor-alpha (TNF-α), interleukin-1 beta (IL-1β), and interleukin-6 (IL-6), thereby exacerbating inflammatory responses (Figure 6A).

AFB1 triggers dose-dependent increases in inflammation and tissue damage, as characterized by enhanced secretion of IL-6 and TNF-α, upregulated NF-κB p65, cyclooxygenase-2 (COX-2), inducible nitric oxide synthase (iNOS), and NOD-like receptor thermal protein domain-associated protein 3 (NLRP3) expression, a significant reduction in serum immune cells, and perturbations of inflammatory lipids and regulator proteins [104]. Transcriptional activation of pro-inflammatory cytokines (*IL-1β*, *IL-6*, and *TNF-α*) and protein-level augmentation of IL-6 and NLRP3 have been corroborated by real-time PCR analysis and immunofluorescence staining [88,105]. Mechanistically, the inflammatory cascades involve the activation of the MAPK and NF-κB pathways, evidenced by increased phosphorylation of both p38 MAPK and NF-κB p65, overexpression of p38 MAPK, ERK1, JNK2, and NF-κB p50, along with the suppression of Nrf2 (an antagonist of NF-κB) [106]. Moreover, AFB1 upregulates NF-κB-associated genes at transcriptional levels, including *NLRP3*, Toll-like receptor 4 (*TLR4*), and thioredoxin-interacting protein (*TXNIP*), potentially forming a self-sustaining inflammatory circuit [107].

Experimental evidence demonstrates that OTA exposure significantly elevates the levels of inflammatory mediators, including NF-κB, TNF-α, IL-1β, and IL-6 [108]. Emerging evidence demonstrates that OTA disrupts intestinal microbiota homeostasis and compromises barrier integrity, triggering systemic inflammation that affects extra-intestinal organs, notably the liver [109]. OTA alters intestinal microbiota diversity and composition, particularly increasing the relative abundance of lipopolysaccharide (LPS)-producing *Bacteroides*, resulting in LPS accumulation and its entrance into the blood and liver through a defective intestinal barrier. After translocation, LPS or the translocated microbiota contributes to inflammation through the involvement of various pattern-recognizing receptors (PRRs). The activation of TLR4 (a special receptor for LPS) leads to the activation of MAPKs and NF-κB pathways, ultimately facilitating the development of liver inflammation [110]. Furthermore, it has been established that OTA induces liver inflammation based on the upregulation of TLR4, myeloid differentiation factor 88 (MyD88), IL-1β, IL-6, TNF-α, and NF-κB p65, though with distinct microbiota compositional alterations compared to previous findings [111].

ZEN significantly elevates pro-inflammatory cytokine levels, such as TNF-α, IL-6, and IL-1β, demonstrating the presence of inflammatory processes [112]. It has also been observed to markedly upregulate the transcription of *IL-6* and *TNF-α*, while decreasing anti-inflammatory *IL-10*. Mechanistically, ZEN promotes the phosphorylation of p38 MAPK and ERK, indicating the engagement of the p38 MAPK/ERK signaling pathway in ZEN-triggered inflammatory responses [113]. Furthermore, ZEN directly compromises the intestinal epithelial barrier and enhances ROS accumulation, which stimulate macrophages to upregulate the transcription of *pro-IL-1β* and *pro-IL-18* [114]. Subsequently, ZEN induces caspase-1 activation via the NLRP3 inflammasome complex, cleaving pro-IL-1β and pro-IL-18 into their biologically active forms, which initiates intestinal inflammatory cascades [114]. Additionally, paralleling OTA effects, ZEN alters the composition of intestinal microbiota, characterized by decreased butyrate-producing bacteria and enhanced LPS-producing bacteria. Reductions in butyrate production lead to intestinal barrier dysfunction, and together with increased LPS in plasma aggravate systemic inflammation [115].

### 3.4. Apoptosis

Apoptosis is a well-studied form of programmed cell death, during which a cascade of caspase activation mediates signal transduction and cellular destruction [116]. It is morphologically characterized by cytosolic shrinkage, plasma membrane blebbing, chromatin condensation, and DNA fragmentation [117]. The present section focuses on elucidating the core molecular machines related to the intrinsic apoptotic pathway.

The BCL-2 protein family constitutes key regulators of intrinsic apoptosis, including pro-apoptotic BCL-2-associated X protein (BAX) and BCL-2 antagonist or killer (BAK), alongside anti-apoptotic BCL-2 proteins, such as BCL-2 and BCL-xL. Intrinsic apoptosis can be triggered by various stimuli, including DNA damage, growth factor withdrawal, mitochondrial damage, and chemotherapeutic agent exposure [116]. Upon activation, BAK/BAX oligomers form pores in the mitochondrial outer membrane (MOM), leading to MOM permeabilization (MOMP) [118]. MOMP enables the release of mitochondrial contents into cytosol, including cytochrome c (cyt c) and second mitochondria-derived activator of caspase (SMAC) [119,120]. Cyt c assembles with apoptotic peptidase activating factor 1 (APAF1) and pro-caspase 9 to form an apoptosome complex that activates the apoptosis initiator caspase-9 [118,120]. Subsequently, caspase-9 cleaves and activates other downstream caspases (e.g., caspase-3 and caspase-7) that can cleave multiple substrates to promote cellular destruction [116]. Additionally, this process can further be amplified by SMAC, which promotes caspase activity by antagonizing inhibitors of apoptosis (IAPs) [117,119] (Figure 6B).

AFB1 provokes DNA fragmentation and leads to apoptotic cell death, which can further be confirmed by the increased apoptosis rates in human hepatocytes [121,122]. The intrinsic apoptotic cascade is amplified through dose-dependently decreased mitochondrial membrane integrity, increased cyt c release into cytoplasm, enhanced caspase-3 activity, transcriptional activation of *caspase-3*, *caspase-9*, and *cyt c*, and concurrent downregulation of *BCL-2* expression [123,124]. Notably, dietary exposure to AFB1 aggravates apoptosis partly by activating p38 MAPK signaling to promote intrinsic apoptosis, which is evidenced by upregulated *p38 MAPK* mRNA level, enhanced expression of pro-apoptotic *APAF1* and *BAX* that are positively correlated with p38 MAPK activation, alongside suppressed anti-apoptotic *BCL-2* and *IAPs* that are negatively correlated with p38 MAPK activation [125].

OTA dose-dependently induces apoptosis through the mitochondrial pathway, evidenced by transcriptional activation of pro-apoptotic mediators (*APAF1*, *cyt c*, *caspase-9*, *caspase-3*, and *BAX*) and suppression of anti-apoptotic *BCL-2*, with corresponding increases in protein levels of APAF1, cleaved-caspase-9, cleaved-caspase-3 and BAX, alongside decreased BCL-xL and BCL-2 [126,127]. Mechanistically, OTA induces apoptosis by activating JNK and p38 MAPK pathways, inducing DNA fragmentation and inhibition of cell cycle progression. It also disrupts cellular component homeostasis, such as that of the mitochondrial membrane, leading to a decrease in both mitochondrial and cytosolic calcium concentrations and consequently causing apoptosis induction [128]. Additionally, OTA-induced intrinsic apoptosis is associated with mitochondrial homeostasis imbalance driven by the downregulation of Lon protease 1 (Lonp1) and tumor necrosis factor receptor-associated protein 1 (TRAP1) [126].

ZEN exposure significantly elevates the proportions of early/late apoptotic cells and induces classical apoptotic hallmarks, such as cyt c release from mitochondria and DNA fragmentation [96,129,130]. Furthermore, ZEN reduces the proportion of S-phase and increases the proportion of sub-G1 in cells, suggesting the occurrence of apoptosis [131]. ZEN upregulates the expression of caspase-9, caspase-3, caspase-7, APAF1, BAX, JNK and p38 MAPK, while also concurrently decreasing anti-apoptotic BCL-2. These results indicate that ZEN aggravates apoptosis partially through the activation of JNK and p38 MAPK signaling pathways [96]. Additionally, the disruption of TNF-α-mediated MAPKK7 and Protein kinase B (AKT) serine/threonine kinase 2 (AKT2) signaling axis participates in ZEN-induced apoptosis, as manifested by increased the mRNA level of *TNF*, and downregulated expression of TNF-downstream *AKT2* and *MAPKK7* [129].

## 4. Nutritional Intervention

Over the past decade, nutritional intervention has emerged as a cornerstone strategy for health enhancement. To address the global challenge of mycotoxin contamination in food systems, this review systematically evaluates two principal countermeasure approaches: utilization of natural and engineered enterosorbents that prevent intestinal absorption and subsequent bioactivation of mycotoxins, and supplementation with dietary small-molecule bioactive compounds that neutralize post-absorption toxicity. In this section, we elucidate the dietary origins, biological activities, and molecular mechanisms of these protective agents, thereby providing scientific evidence for developing multi-component therapeutic strategies against mycotoxin-related health issues.

### 4.1. Natural and Engineered Enterosorbents

**Figure 7:**
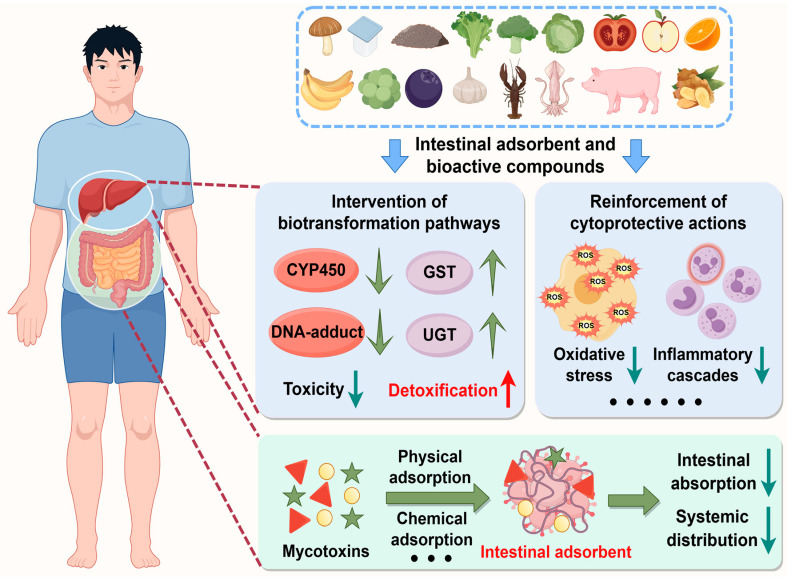
Synergistic protective mechanisms of nutritional intervention through enterosorbent-mediated absorption blockade and bioactivation suppression, along with bioactive compound-driven biotransformation intervention and cytoprotective actions.

Mycotoxin enterosorbents are a class of specialized compounds that adsorb toxins onto their surfaces and prevent their intestinal absorption and following entrance into systemic circulation [132]. This part evaluates the application potential of mycotoxin enterosorbents as functional food and feed additives through a source-specific classification system, focusing on three primary categories: microbial-derived materials (e.g., edible fungi, lactic acid bacteria, and yeasts), bio-based polymeric substances (e.g., cellulose, lignin, and chitosan), and inorganic–organic hybrid materials (e.g., modified clinoptilolite and montmorillonite) (Figure 7).

As evidenced by both in vitro co-culture systems and in vivo evaluations, *Auricularia auricula* exerts hepatoprotective effects against AFB1-induced liver injury, which can be attributed to the reduction in AFB1 bioavailability through both physical and chemical adsorption, attenuation of oxidative stress, and augmentation of phase-II enzyme activity [133]. The Kefir consortium exhibits efficient mycotoxin adsorption capabilities in vitro co-culture conditions, demonstrating considerable removal of AFB1, OTA, and ZEN. While microorganism–mycotoxin complexes are partially reversible under gastrointestinal conditions, the retained binding capacity suggests that Kefir supplementation may help to reduce mycotoxin gastrointestinal absorption [134]. It was assumed that that bacterial polysaccharides may be primarily responsible for the observed adsorption effects [134]. Through in vitro adsorption assays of AFB1 in functional yogurts, the combined use of 1.0–4.0% inactivated biomasses of *Lacticaseibacillus rhamnosus* (LRB) and *Saccharomyces cerevisiae* (SCB) enhances AFB1 adsorption efficiency to 86.9–91.2%. Although the precise mechanisms require further elucidation, the adsorption process may involve the binding to cell wall components, hydrophobic and electrostatic interactions, and non-covalent interactions. Notably, the increased cell survival following exposure to yogurts containing ≥1% LRB + SCB indicates potential protective effects against AFB1-induced cytotoxicity [135].

Blackberry seed oil cake (BBSOC) flour, a significant source of dietary fiber (62.09% dry weight) and essential minerals, demonstrates effective adsorption of AFB1 with adsorption efficiencies of 85.4% and 87.0% at pH 3 and 7, respectively. The immobilization mechanisms of AFB1 are primarily attributed to carboxyl and hydroxyl groups on the biosorbent surface, which bind AFB1 through physicochemical interactions and complexation [136]. Furthermore, lignins from *Rhododendron tomentosum* and *Althaea officinalis* exhibit superior AFB1 adsorption capabilities, achieving irreversible adsorption indices of 75.4% and 79.2%, respectively. Chemisorption plays the most important role in these adsorption processes, involving the formation of hydrogen bonds with acidic hydroxyl groups of lignins [137]. Compared with cellulosic polymers, high-molecular-weight and non-cross-linked chitosan shows moderate adsorbent capacities for AFB1 (37.5%), OTA (50.6%), and ZEN (75.6%). Further modifications, such as cross-linking, deacetylation degree optimization, and molecular weight adjustment, may be helpful to enhance its overall adsorption performance and broaden its applicability in mycotoxin mitigation strategies [138].

Modified clinoptilolite (MZ) is an adsorbent developed through an ion exchange reaction between inorganic cations on the mineral surface and organic cations, which results in the formation of new active centers on the mineral surface. This modification facilitates the efficient binding of both polar mycotoxins (e.g., AFs) and non-polar mycotoxins (e.g., OTA and ZEN). The supplementation of 0.2% MZ in laying hen feed mitigates OTA toxicity by enhancing production performance, improving histopathological alterations, and reducing OTA residue levels in eggs [139]. In single systems, nonionic surfactant octylphenol polyoxyethylene ether (OP-10)-modified montmorillonites (NMts) significantly improve adsorption capacities for AFB1 and ZEN, increasing from 0.5 mg/g and 0.0 mg/g (raw Mt) to 2.8 mg/g and 8.5 mg/g, respectively. The adsorption mechanisms of NMts to AFB1 involve both ion–dipole interaction and hydrophobic interaction, whereas ZEN adsorption is hydrophobic interaction [140]. In binary-contaminant systems, competitive adsorption has been observed, where AFB1 significantly suppresses ZEN uptake due to its stronger affinity via ion–dipole forces, while ZEN has little effect on AFB1 adsorption. Notably, NMts exhibit certain adsorption efficiencies at low surfactant loadings, highlighting their promising use as a cost-effective and versatile adsorbent for the simultaneous detoxification of polar and non-polar mycotoxins [140].

### 4.2. Dietary Small-Molecule Bioactive Compounds

Dietary small-molecule bioactive compounds play significant roles in alleviating mycotoxin toxicity following intestinal absorption, offering crucial protection against toxin-related issues (Table S3). This section specifically focuses on four representative classes: flavonoids, phenolic acids, sulfur-containing organic compounds, and other promising natural agents. The detoxification mechanisms function through two synergistic axes: intervention of biotransformation pathways that primarily involve the suppression of phase-I metabolic activation and the enhancement of phase-II clearance processes, as well as reinforcement of cytoprotective actions including alleviating redox imbalance, suppressing inflammatory responses, modulating apoptotic pathways, and interfering with cancer progression. Collectively, these mechanisms establish an integrated defensive network against mycotoxin-mediated cytotoxic stress (Figure 7).

#### 4.2.1. Flavonoids and Their Derivates

Flavonoids, constituting the predominant class of polyphenolic secondary metabolites, are ubiquitously distributed in various food sources, including fruits, vegetables, nuts, cereals, seeds, and tea [141,142]. In this section, we highlight the protective roles of quercetin (QUE) along with its rhamnoside derivative quercitrin (QUN), apigenin (API), proanthocyanidins (PAs), and catechin (CATE).

QUE supplementation exhibits inhibitory effects on *CYP1A1* and *CYP3A4* expression, suppresses CYP3A activities, while upregulating GSTA1 and then increasing GSH levels [143]. API has been observed to inhibit CYP1A2 activity and reinforce the activity of GST and the levels of GSH and total sulfhydryl groups (TSHs) [144]. Moreover, PAs significantly reduce the concentration of hepatic AFB1 residues, suggesting their potential roles in promoting hepatic detoxification pathways [11]. Molecular docking simulations reveal that CATE and QUN can interact with CYP1A2 and CYP3A4, indicating their potential to competitively inhibit AFB1 binding, block its metabolic activation, and consequently diminish the generation of toxic intermediates [145]. Additionally, flavonoid compounds may modulate toxin biotransformation through indirect pathways. For instance, QUE increases the relative abundance of *Akkermansia muciniphila*, which produces more indole-3-lactic acid to upregulate the expression of 12*α*-hydroxylase (CYP8B1), thereby promoting the conversion of cholesterol to cholic acid (CA) [146]. CA subsequently activates the farnesoid X receptor (FXR), which mediates the activation of UGT1A3 and then promotes phase-II glucuronidation reactions [147].

Previous research has elucidated further cytoprotective mechanisms associated with flavonoids. API exhibits strong radical scavenging activity, underscoring its potential as a natural antioxidant. Moreover, under food thermal processing, API can be transformed into ester derivatives with enhanced lipophilicity and cellular antioxidant activity [148]. Furthermore, PAs from Kiwi Leaves (*Actinidia chinensis*) activate the Keap1-Nrf2 pathway through competing with Nrf2 to promote its separation from the inactive complexes. This action significantly upregulates the expression levels of downstream antioxidant genes, including *HO-1*, *NQO1*, *SOD-1*, and *CAT*, thereby effectively improving cellular antioxidant status [149]. QUE and CATE exhibit synergistic anti-inflammatory effects via the suppression of TLR4-MyD88-mediated NF-κB and MAPK signaling pathways. The mechanisms involve downregulation of MyD88 expression at both transcription and protein levels, blockade of NF-κB nuclear translocation through inhibiting p65/p50 subunit transcription and phosphorylation, and suppression of ERK, JNK, and p38 MAPK phosphorylation. Such alterations collectively inhibit the production of pro-inflammatory mediators and cytokines [150]. Bioactive compounds exhibit bifunctional apoptotic modulation in a pathology-dependent manner. For instance, QUE inhibits mitochondrial apoptotic pathway, as it significantly improves the loss of MMP, decreases cyt c levels, suppresses the activities of caspase-3 and caspase-9, and downregulates the ratios of BAX/BCL-2 [151]. Regarding the anticancer activity, API induces apoptosis, autophagy, and cell cycle arrest through the regulation of various signaling pathways involved in cancer progression, such as the phosphatidylinositol-3-kinase (PI3K)/Akt/mammalian target of rapamycin (mTOR), Janus Kinases (JAK)/Signal Transducer and Activator of Transcription (STAT), NF-κB, and MAPK/ERK signaling pathways. Additionally, the dual chemo/radiosensitization and chemo/radioprotection activities contribute to its multidimensional anticancer efficacy [152].

#### 4.2.2. Phenolic Acids and Their Derivates

Phenolic acids, a crucial subclass of phenolic compounds, are widely present in various plant sources such as fruits, vegetables, spices, grains and beverages. As aromatic secondary metabolites, they impart color, flavor, astringency, and harshness, contributing to the typical organoleptic characteristics of the foods [153]. Key representatives of phenolic acids and their derivates include ferulic acid (FA), *p*-coumaric acid (CA), gallic acid (GA), chlorogenic acid (CGA), and its special isomer cryptochlorogenic acid (CCGA).

FA effectively blocks AFB1 metabolic activation by inhibiting the relative protein expression of CYP1A2, CYP2A6, CYP3A4, and CYP2E1, while also competing with AFB1 for CYP2A6. Meanwhile, FA accelerates the detoxification processes by enhancing GST activity, and upregulating the mRNA expression of *GSTA3*, *GSTA5*, and *GSTM2*. Additionally, a reduction in liver AFB1-DNA adducts and serum AFB1-ALB adduct levels has been observed, further supporting the protective role of FA [45,154]. CA from whole highland barley significantly increases the relative mRNA expression of *SULT1A1*, *UGT1A1*, *UGT1A4*, and *UGT1A6*, probably indicating its capacity to strengthen phase-II sulfation and glucuronidation processes [155]. GA elicits a marked elevation in GSH level and total cellular thiol content, further promoting the conjugation reactions and decreasing the bioaccumulation of harmful intermediates [156]. CGA has been demonstrated to augment hepatic GSH level, while its special isomer CCGA elevates intracellular total GSH and GSH/GSSG (oxidized GSH) ratio in a dose-dependent manner, collectively improving tissue detoxification and antioxidant capacity [157,158].

FA exerts potential antioxidant effects through its phenolic hydroxyl group, which donates electrons to quench free radicals [159]. It also elevates the relative protein expression of Nrf2, HO-1, NQO1, GCLC, and GPx4, while downregulating the expression of Keap1, suggesting that FA may enhance cellular antioxidant capacity by stimulating downstream antioxidant enzymes through the activation of the Keap1-Nrf2 signaling pathway [159,160]. CA directly binds to NOX2 and suppresses the production of ROS in hepatocytes, thus preventing cell death and liver injury [161]. GA inhibits the activities of MAPK and NF-κB, subsequently suppressing the release of inflammatory mediators, including TNF-α, IL-1β, IL-6, intercellular adhesion molecule 1 (ICAM-1), COX-2, and nitric oxide (NO). It also decreases inflammatory cell infiltration, thereby ameliorating the inflammatory response [162]. As an effective natural anticancer agent officially approved by the China Food and Drug Administration, CGA demonstrates broad-spectrum anticancer efficacy against diverse malignancies, including leukemia and breast, colorectal, esophageal, hepatic, lung cancer, etc. This therapeutic potential primarily originates from cell cycle arrest, induction of apoptosis, and suppression of cancer cell proliferation, migration, and invasion [163,164].

#### 4.2.3. Sulfur-Containing Organic Compounds

Biologically active sulfur-containing organic compounds include representative compounds such as isothiocyanates (e.g., sulforaphane [SFN] and phenylethyl isothiocyanate [PEITC]), allicin, taurine, and GSH. These phytochemicals can be primarily found in specific dietary sources, including cruciferous vegetables (e.g., broccoli and cauliflower), garlic, animal-derived products (especially marine organisms and mammals), and fruits (e.g., oranges, apples, and bananas) [165,166,167,168] (Figure 7).

SFN decreases the mRNA expression of *CYP1A2* and *CYP3A4*, leading to a significant reduction in DNA adduct formation [169]. The significantly increased hepatic GST activity and augmented expression of the representative phase-II enzyme UGT have also been observed, further suggesting the therapeutic effects of SFN [170,171]. Furthermore, PEITC not only suppresses the transcriptional expression of *CYP3A4* and *CYP3A5* in primary human hepatocyte cultures, but also inhibits the catalytic activities of both CYP1A2 and CYP3A4, thereby moderately decreasing AFB-DNA adduct formation [169]. Allicin suppresses the activity of CYP2E1 by binding to its amino acid residues Phe116, Phe207, Leu210, Phe298, Ala299, Thr303, Val364, and Phe478 through hydrophobic interactions, while also concurrently decreasing CYP2E1 protein expression [172]. In addition, garlic powders with various levels of alliin—which can be metabolized to allicin by alliinase upon subjected to cutting, chopping, or squeezing—lead to increased hepatic GST and UGT activities, alongside augmented levels of GSTA5 and AFAR [173]. Taurine supplementation significantly increases serum GSH concentration while simultaneously decreasing GSSG level. It also elevates the activity of glutathione synthase (GSHS), which is responsible for GSH synthesis. This effect originates from the shared cysteine precursors involved in both taurine and GSH synthesis. Exogenous taurine consumption reduces its endogenous synthesis, thereby redirecting a lot of cysteine for GSH production. Consequently, the saved cysteine activates GSHS as a substrate, thus synthesizing more GSH for mycotoxin detoxification [174]. The consumption of fruits and vegetables containing good concentrations of GSH may not directly elevate innate GSH level but instead promote its endogenous intracellular biochemical production, thereby enhancing the metabolic detoxification processes [168].

SFN activates the Keap1-Nrf2 signaling pathway through two distinct molecular mechanisms, thereby promoting the induction of various cytoprotective enzymes with detoxifying and antioxidant action. The primary mechanism involves the modification of Keap1 cysteine residues, which facilitates the release of Nrf2 and its subsequent nuclear translocation. Moreover, SFN induces epigenetic modulation of HDACs and DNA methyltransferases (DNMTs), thus enhancing Nrf2 transcription and translation [175]. Taurine also enhances the antioxidant capacity, as manifested by decreased MDA concentration, increased ROS clearance rate, augmented T-AOC, and elevated serum activities of SOD, GPx, CAT, and peroxidase, alongside the upregulated mRNA abundances of *HO-1*, *GPx1*, *SOD1*, and *SOD2* [174]. Furthermore, SFN blocks the phosphorylation of ERK1/2, JNK, p38 MAPK, and NF-κB p65, thus suppressing the expression of TNF-α, IL-1β, IL-6, and iNOS [176]. Taurine attenuates apoptosis in piglet jejunum by reversing the upregulated expression of pro-apoptotic genes, including *BAX*, *cyt c*, *caspase-9*, *caspase-3*, and *AIFM1*, alongside the downregulated expression of *BCL2* [177]. Due to its reactive nature and capacity to interact with multiple molecular targets, allicin astonishingly exerts the ability to counteract all phases of carcinogenesis. The suppression of DNA damage, inhibition of cell proliferation, angiogenesis, and metastatic processes, along with the induction of cell death, collectively contribute to the antitumor effects of allicin [178].

#### 4.2.4. Other Promising Natural Agents

Beyond the three categories previously mentioned, emerging research highlights the significance of additional dietary small-molecule bioactive compounds, including lycopene (LYC), astaxanthin (ASTA), curcumin (CUR), and resveratrol (Res). These compounds have demonstrated multi-target efficacy in mitigating mycotoxin-induced toxicities. LYC belongs to the carotenoid family and is abundant in watermelons, guavas, grapefruits, apricots, papayas, and notably, tomatoes [179]. It has been identified as A-grade nutrient by the FAO, Joint Expert Committee on Food Additives (JECFA), and World Health Organization (WHO) [180]. ASTA, a lipid-soluble marine carotenoid, is primarily sourced from algae, yeast, trout, red seabream, and the waste of crustaceans such as shrimp and crabs [181]. CUR is a natural hydrophobic polyphenol compound derived from the roots/rhizomes of the turmeric plant (*Curcuma longa*) [182,183]. Res is a bioactive polyphenolic phytochemical widely distributed in grapes, wine, peanuts, mulberries, blueberries, and strawberries [184] (Figure 7).

LYC supplementation inhibits the activities of CYP1A1, CYP2A6, and CYP2E1 enzymes in the liver, thereby reducing the formation of AFBO, AFP1, AFM1, AFQ1, and AFB1-DNA adducts. Moreover, it upregulates the levels of GSH and phase-II enzyme GSTs, enhances the production of AFBO-GSH and AFB1-dialcohol, and ultimately facilitates the detoxification of AFB1 [185]. ASTA decreases the mRNA expression levels of NRs (*ahr*, *pxr*, and *car*) and CYP450 (*cyp1a1*, *cyp1a5*, *cyp2c18*, *cyp2d6*, and *cyp3a9*), thereby effectively inhibiting phase-I metabolic responses [40]. Additionally, it may promote GSH levels and upregulate the expression of GCLC and GPx for GSH synthesis [90]. CUR enhances the overall DNA methylation level and the expression of DNMTs (*DNMT1*, *DNMT3a*, and *DNMT3b*), which subsequently decrease the expression and activity of hepatic CYP450 enzymes (*CYP1A1*, *CYP1A2*, and *CYP3A4*) [186]. Additionally, dietary CUR has been demonstrated to downregulate *CYP2A6* expression, while inducing the expression of phase-II enzymes (*GSTA3* and *GSTM2*) and enhancing GST enzyme activity [187]. Res supplementation significantly decreases the total CYP450 content and the expression levels of *CYP1A1* and *CYP3A4*, while increasing GSH content, GST activity, and the expression of *GST*, *GCLC*, and *UGT1A8* [188,189].

Notably, ASTA exhibits the strongest antioxidant ability among carotenoids, a property attributed to its molecular structure that enables it to effectively quench singlet oxygen and scavenge free radicals. It promotes the nuclear translocation of Nrf2, which activates downstream cytoprotective targets including *NQO1*, *HO-1*, and *GPx*, collectively improving cellular antioxidant capacity [94]. Furthermore, amongst different carotenoids, LYC is recognized as the most potent antioxidant, second only to ASTA [190]. It activates Nrf2 nuclear translocation to induce the expression of the downstream genes *HO-1* and *NQO1* and elevates the contents of antioxidant enzymes (GPx, SOD, and T-AOC) [191]. CUR-containing prepared nanoparticles have been observed to suppress the expression and nuclear translocation of phospho-NF-κB p65, leading to a decrease in pro-inflammatory mediator production [192,193]. Res likely inhibits JNK and p38 MAPK signaling pathways through reducing ROS accumulation, thereby suppressing the inflammatory response, as evidenced by the downregulated levels of pro-inflammatory cytokines such as TNF-α, IL-1β, IL-6, and monocyte chemoattractant protein-1 (MCP-1). Concurrently, the blockade of JNK activation also contributes to inhibiting cell proliferation and inducing apoptosis [194]. ASTA also acts against cell apoptosis by blocking p-ERK/ERK, cyt c, caspase-3, caspase-9, and the BAX/BCL-2 ratio [195]. LYC demonstrates potential anticancer effects through its capacity to quench singlet oxygen, induce cytoprotective enzyme production, initiate apoptosis, and inhibit cell proliferation and cell cycle progression, as well as modulate gap junctional communication, growth factors, and signal transduction pathways [196].

## 5. Conclusions

Mycotoxins, as secondary fungal metabolites, pose significant contamination risks in food/feed commodities and TCMs, thereby jeopardizing global food security and incurring considerable economic losses. Among more than 400 mycotoxins identified, AFB1, OTA, and ZEN are of particular concern due to their widespread occurrence and high toxicity. These toxins enter the human body via contaminated food chains, causing acute poisoning or chronic cumulative damage, thereby posing significant health risks.

Initially, a comprehensive understanding of mycotoxin biosynthetic pathways and their in vivo biotransformation is essential for the prevention, control, and resolution of issues related to mycotoxins. In the context of biosynthetic pathways, Table S2 presents the genes and their associated functions that are involved in mycotoxin synthesis. Furthermore, we elucidate the major biosynthetic processes observed in representative strains. Regarding the biotransformation pathways, phase-I metabolic activation generates reactive intermediates that disrupt cellular homeostasis and contribute to toxic effects. In contrast, phase-II biotransformation reactions are typically regarded as detoxification processes, which involve conjugation with nucleophilic molecules such as GSH, sulfate, and glucuronic acid. Mycotoxin prototypes, along with reactive metabolites and by-products (such as ROS) generated during metabolic processes, may lead to various adverse effects through mechanisms associated with carcinogenesis, inhibition of protein synthesis, estrogenic effects, oxidative stress, inflammation, and abnormal apoptosis, collectively threatening human and livestock health. Notably, the application of natural and engineered adsorbents may block the intestinal absorption and subsequent metabolic activation of mycotoxins. Following absorption, dietary supplementation with small-molecule bioactive compounds may counteract mycotoxin toxicity via suppression of phase-I metabolic bioactivation and enhancement of phase-II clearance processes. Concurrently, they also reinforce cytoprotective actions by maintaining redox homeostasis, inhibiting inflammatory cascades, modulating apoptotic pathways, and interfering with cancer progression.

In conclusion, this review systematically elucidates the biosynthetic pathways, biotransformation fates, and pathogenic mechanisms of AFB1, OTA, and ZEN, while evaluating the therapeutic potential of nutritional intervention. It is anticipated that this review will furnish valuable insights to researchers, advancing the studies on mycotoxin toxicity and the development of dietary countermeasures against mycotoxin-induced damage.

## Data Availability

No data was used for the research described in the article.

## Supplementary Materials

The following supporting information can be downloaded at: https://www.mdpi.com/article/10.3390/molecules30193860/s1. Figure S1. Schematic representation of AFB1 biosynthetic pathways and involving functional genes; Table S1. Summary of the principal mycotoxins, including their corresponding fungal producers, the foods susceptible to contamination, their toxicological effects, and classifications by the International Agency for Research on Cancer (IARC); Table S2. Summary of genes involved in the biosynthesis of AFB1, OTA, and ZEN, including the old and new cluster gene nomenclatures, alongside their functional roles in representative fungal species; Table S3. Summary of the cytoprotective effects of dietary small-molecule bioactive compounds, including metabolic intervention and broad-spectrum protection.

## Abbreviations

The following abbreviations are used in this manuscript:

AFs	aflatoxins
OTs	ochratoxins
ZEN	zearalenone
TCMs	traditional Chinese medicines
FAO	Food and Agriculture Organization of the United Nations
AFB1	aflatoxin B1
OTA	ochratoxin A
IARC	International Agency for Research on Cancer
ERs	estrogen receptors
NAA	norsolorinic acid anthrone
NOR	norsolorinic acid
AVN	averantin
HAVN	5′-hydroxyl-averantin
OAVN	5′-oxoaverantin
AVF	averufin
HVN	hydroxyversicolorone
VHA	versiconal hemiacetal acetate
VAL	versiconal
VOAc	versiconol acetate
VOH	versiconol
VERB	versicolorin B
VERA	versicolorin A
DMST	demethylsterigmatocystin
ST	sterigmatocystin
OMST	*O*-methylsterigmatocystin
CYP450	cytochrome P450
NR	nuclear receptor
AHR	aryl hydrocarbon receptor
CAR	constitutive androstane receptor
PXR	pregnane X receptor
GSH	glutathione
GSTs	glutathione-S-transferases
mEH	microsomal epoxide hydrolase
AFAR	aflatoxin–aldehyde reductase
AFBO	AFB1-exo-8, 9-epoxide
AFL	aflatoxicol
AFM1	aflatoxin M1
AFP1	aflatoxin P1
AFQ1	aflatoxin Q1
AFH1	aflatoxicol H1
AFB2a	aflatoxin B2a
GGT	γ-glutamyltranspeptidase
DPEP	dipeptidase
NAT	N-acetyltransferase
AFB1-NAC	AFB1-mercapturic acid
dG	deoxyguanosine
AFB1-FAPY-dG	AFB1-formamidopyrimidine-dG
AP	abasic site
AFB1-ALB	AFB1-albumin
AFB1-dhd	AFB1-8,9-dihydrodiol
FAD	flavin adenine dinucleotide
PKS	polyketide synthase
OT*β*	ochratoxin *β*
NRPS	nonribosomal peptide synthase
Phe	L-*β*-phenylalanine
OT*α*	Ochratoxin *α*
OP-OA	lactone-opened OTA
OTQ	OTA-quinone
OTHQ	OTA-hydroquinone
*β*-ZEL	*β*-zearalenol
HSDs	3*α*-/3*β*-hydroxysteroid dehydrogenases
*α*-ZEL	*α*-zearalenol
*α*-ZAL	*α*-zearalanol
*β*-ZAL	*β*-zearalanol
ZAN	zearalanone
UGT	UDP glucuronosyltransferases
SULTs	sulfotransferases
ZEN-14-*O*-G	ZEN-14-*O*-glucuronide
8-OHdG	8-hydroxydeoxyguanosine
HAT	histone acetyl transferase
HDAC	histone deacetylase
PheRS	phenylalanine tRNA synthase
PAH	phenylalanine hydroxylase
EREs	estrogen response elements
ROS	reactive oxygen species
ETC	electron transport chain
ER	endoplasmic reticulum
NOXs	NADPH oxidases
mtDNA	mitochondrial DNA
MMP	mitochondrial membrane potential
mPTP	mitochondrial permeability transition pore
Keap1	Kelch-like ECH-associated protein 1
Nrf2	nuclear factor-E2-related factor 2
Cul3	Cullin 3
sMAF	small musculo-aponeurotic fibrosarcoma
AREs	antioxidant responsive elements
GCL	glutamate-cysteine ligase
NQO1	NAD(P)H oxidoreductase 1
HO-1	heme oxygenase-1
GPx	glutathione peroxidase
CAT	catalase
SOD	superoxide dismutase
MDA	malondialdehyde
KEGG	Kyoto Encyclopedia of Genes and Genomes
MAPK	mitogen-activated protein kinase
NF-κB	nuclear factor kappa-B
ERK	extracellular signal-regulated protein kinase
JNK	c-Jun amino (N)-terminal kinase
IKK	IκB kinase
TNF	tumor necrosis factor
IL	interleukin
COX-2	cyclooxygenase-2
iNOS	inducible nitric oxide synthase
NLRP3	NOD-like receptor thermal protein domain associated protein 3
TLR4	Toll-like receptor 4
TXNIP	Thioredoxin-interacting protein
LPS	lipopolysaccharides
PRRs	pattern-recognizing receptors
MyD88	myeloid differentiation factor 88
MOM	mitochondrial outer membrane
cyt c	cytochrome c
SMAC	second mitochondria-derived activator of caspase
APAF1	apoptotic peptidase activating factor 1
IAPs	inhibitors of apoptosis
LRB	*Lacticaseibacillus rhamnosus*
SCB	*Saccharomyces cerevisiae*
QUE	quercetin
QUN	quercitrin
API	apigenin
PAs	proanthocyanidins
CATE	catechin
TSH	total sulfhydryl group
PI3K	phosphatidylinositol-3-kinase
mTOR	mammalian target of rapamycin
JAK	Janus Kinases
STAT	Signal Transducer and Activator of Transcription
FA	ferulic acid
CA	*p*-coumaric acid
GA	gallic acid
CGA	chlorogenic acid
CCGA	cryptochlorogenic acid
ICAM-1	intercellular adhesion molecule 1
NO	nitric oxide
SFN	sulforaphane
PEITC	phenylethyl isothiocyanate
GSHS	glutathione synthase
DNMTs	DNA methyltransferases
LYC	lycopene
ASTA	astaxanthin
CUR	curcumin
Res	resveratrol
JECFA	Joint Expert Committee on Food Additives
WHO	World Health Organization
