# Supplementary material for: Mycotoxin Contamination: Occurrence, Biotransformation, Pathogenic Mechanisms, and Strategies for Nutritional Intervention
Source: Molecules. 2025 Sep 23;30(19):3860. doi: 10.3390/molecules30193860 (PMC12525999; doi:10.3390/molecules30193860)
Supplement: Supplementary file 1 [file molecules-30-03860-s001.zip › molecules-3849415-supplementary.pdf]

Mycotoxin Contamination: occurrence, biotransformation, pathogenic mechanisms, and strategies for nutritional intervention

Chenyu Yao [a], Mengyu Ye [a], Cong Wang [a], Lin Zou [a], Ximeng Zhang [a], Xin Chai [a, b], Huijuan Yu [a, b], Chengyu Zhang [a, b, *], Yuefei Wang [a, b, *]

[*]Corresponding authors:

Chengyu Zhang (zhangchengyu_ok@126.com); Yuefei Wang (wangyf0622@tjutcm.edu.cn)

[a] State Key Laboratory of Chinese Medicine Modernization, Tianjin Key Laboratory of TCM Chemistry and Analysis, Tianjin University of Traditional Chinese Medicine, Tianjin 301617, China.

[b] Haihe Laboratory of Modern Chinese Medicine, Tianjin 301617, China.

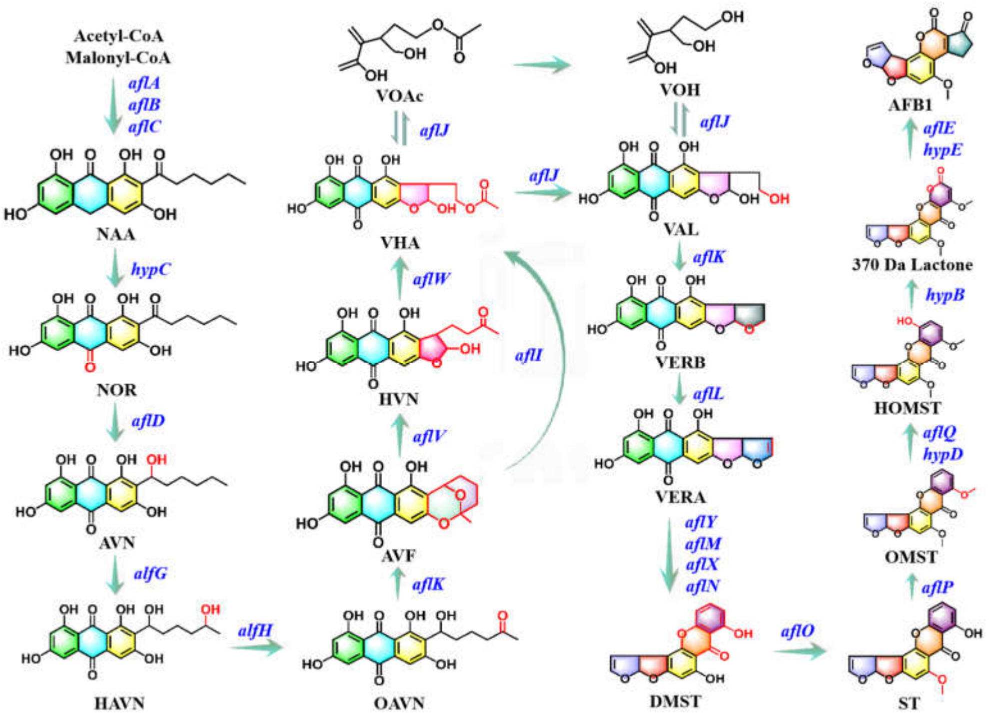

Figure S1 Schematic representation of AFB1 biosynthetic pathways and involving functional genes.

Table S1 Summary of the principal mycotoxins, including their corresponding fungal producers, the foods susceptible to contamination, their toxicological effects, and classifications by the International Agency for Research on Cancer (IARC).

**mycotoxins**	**primary fungal producers**	**susceptible foods**	**toxicological effects**	**IARC classification**	**references**
Aflatoxin B1 (AFB1)	*Aspergillus flavus* and *Aspergillus parasiticus*	crops, such as maize, wheat, peanuts, and rice	hepatotoxicity, immunotoxicity, carcinogenicity, teratogenicity, and mutagenicity	Group I	[1, 2]
Ochratoxin A (OTA)	*Aspergillus ochraceus* and *Penicillium verrucosum*	food products, beverages, and animal-derived products	nephrotoxicity, hepatotoxicity, mutagenicity, teratogenicity, and immunotoxicity	Group IIB	[3-5]
Zearalenone (ZEN)	*Fusarium graminearum* and *Fusarium culmorum*	grains (such as corn, wheat, rice, barley, and sorghum) and animal milk	reproductive toxicity, immunotoxicity, teratogenicity, carcinogenicity, and genotoxicity	Group III	[6-8]
Deoxynivalenol	*F. graminearum*	grains and feeds, such as wheat, barley, and corn	anorexia, emesis, weight loss, disorders in neuroendocrine, and immunotoxicity	Group III	[9, 10]
Fumonisin B1	*Fusarium proliferatum* and *Fusarium verticillioide*	corn and corn products, rice and other cereals (such as oat, rye, barley, and wheat	immunotoxicity, hepatotoxicity, nephrotoxicity, and reproductive toxicity	Group IIB	[11, 12]
Citrinin	*Penicillium citrinum*, *Penicillium expansum*, *P. verrucosum*, *Aspergillus niveus*, *Aspergillus terreus*, and *Monascus ruber*	food and foodstuff, grains such as corn, wheat, rye, barley, oats and rice	nephrotoxicity, hepatotoxicity, embryocidal and fetotoxic effects	Group III	[13]

Table S2 Summary of genes involved in the biosynthesis of AFB1, OTA, and ZEN, including the old and new cluster gene nomenclatures, alongside their functional roles in representative fungal species.

Mycotoxin	Fungal specie	Biosynthetic gene	Putative role	References
AFB1	*A. flavus and A. parasiticus*	*aflA (fas-2)*	Fatty acid synthase α	[14]
		aflB (fas-1)	Fatty acid synthase β	[14]
		aflC (pksA)	Polyketide synthase (PKS)	[15]
		aflD (nor-1)	Ketoreductase	[15]
		aflE (norA)	Alcohol dehydrogenase	[15]
		aflG (avnA)	P450 monooxygenase	[16]
		aflH (adhA)	Alcohol dehydrogenase	[16]
		aflI (avfA)	Oxidoreductase	[15]
		aflJ (estA)	Esterase	[14]
		aflK (vbs)	Vesicolorin B synthase	[15]
		aflL (*verB*)	cytochrome P450 monooxygenase	[14]
		aflM (ver-1)	Ketoreductase	[14]
		aflN (verA)	cytochrome P450 monooxygenase	[14]
		aflO (omtB)	O-methyltransferase B	[16]
		aflP (omtA)	O-methyltransferase A	[16]
		aflQ (ordA)	Cytochrome P450 monooxygenase	[15]
		aflR	Transcription activator	[15]
		aflS (*aflJ*)	Pathway co-regulator	[15]
		aflV (cypX)	Cytochrome p450 monooxygenase	[15]
		aflW (moxY)	Monooxygenase	[15]
		aflX (ordB)	Oxidoreductase	[14]
		aflY (hypA)	Baeyer-Villiger oxidase	[17]

		hypB (hypB2)	Oxidase	[15]
		hypC (hypB1)	Oxidase	[15]
		hypD	Integral membrane protein	[15]
		hypE (aflLa)	EthD family protein	[15]
OTA	*A. ochraceus*	*otaA*	Polyketide synthase (PKS)	[18]
		otaB	Nonribosomal peptide synthetase (NRPS)	[18]
		otaC	Cytochrome P450 monooxygenase	[18]
		otaD	Halogenase	[18]
		otaE	Flavin adenine dinucleotide (FAD)-dependent oxidoreductase	[18]
		otaR1	Transcription activator	[18]
		otaR2	Pathway co-regulator	[18]
		otaY	Cyclase	[19]
ZEN	*F. graminearum*	*PKS4*	Reducing PKS	[20]
		PKS13	Non-reducing PKS	[20]
		ZEB1	Isoamyl alcohol oxidase	[20]
		ZEB2	a basic leucine zipper (bZIP) transcription factor	[15]

Table S3 Summary of the cytoprotective effects of dietary small-molecule bioactive compounds, including metabolic intervention and broad-spectrum protection.

Category	Function	Compound	Mechanisms	References
Flavonoids	Metabolic intervention	QUE	↓*CYP1A1* and *CYP3A4* expression; CYP3A activities;<br>↑GSTA1; GSH levels	[21]
		API	↓CYP1A2 activity;<br>↑GST activity; GSH and TSH levels	[22]
		PAs	↓hepatic AFB1 residues	[23]
		CATE and QUN	interact with CYP1A2 and CYP3A4→ ↓phase-I metabolic activation	[24]
		QUE	↑relative abundance of *Akkermansia muciniphila*→ ↑UGT1A3	[25, 26]
	Cytoprotective actions	API	exhibit strong ABTS and DPPH radical scavenging activity	[27]
		PAs	activate Keap1-Nrf2 pathway;<br>↑*HO-1*, *NQO1*, *SOD-1*, and *CAT* expression	[28]
		QUE and CATE	suppress TLR4-MyD88-mediated NF-κB and MAPK signaling pathways;<br>↓pro-inflammatory mediators production	[29]
		QUE	inhibit mitochondrial apoptotic pathway;<br>↓MMP loss; cyt *c* levels; caspase-3 and caspase-9 activities; ratios of BAX/BCL-2	[30]
		API	exhibit anticancer activity;<br>induce apoptosis; autophagy; and cell cycle arrest;<br>chemo/radiosensitization and chemo/radioprotection	[31]

Phenolic acids	Metabolic intervention	FA	↓*CYP1A2*, *CYP2A6*, *CYP3A4*, and *CYP2E1* expression;<br>↓liver AFB1-DNA adducts and serum AFB1-ALB adduct levels;<br>compete with AFB1 for CYP2A6 enzyme;<br>↑GST activity; *GSTA3*, *GSTA5*, and *GSTM2* expression	[32, 33]
		CA	↑*SULT1A1*, *UGT1A1*, *UGT1A4*, and *UGT1A6* expression	[34]
		GA	↑GSH level; total cellular thiols content	[35]
		CGA	↑hepatic GSH level	[36]
		CCGA	↑intracellular total GSH; GSH/GSSG ratio	[37]
	Cytoprotective actions	FA	donate electrons to quench free radicals (phenolic hydroxyl group);<br>activate Keap1-Nrf2 pathway;<br>↑*Nrf2*, *HO-1*, *NQO1*, *GCLC*, and *GPx4* expression;<br>↓*Keap1* expression	[38, 39]
		CA	directly bind to NOX2 and suppress ROS production	[40]
		GA	↓MAPK and NF-κB activities;<br>↓inflammatory mediators release, including TNF-α, IL-1β, IL-6, ICAM-1, TIMP-1, COX-2, and NO;<br>↓inflammatory cell infiltration	[41]
		CGA	cell cycle arrest, induction of apoptosis, and suppression of cancer cell proliferation, migration, and invasion	[42]
Sulfur-containing organic compounds	Metabolic intervention	SFN	↓*CYP1A2* and *CYP3A4* expression; DNA adduct formation<br>↑hepatic GST activity; *UGT* expression	[43-45]
		PEITC	↓*CYP3A4* and *CYP3A5* expression; CYP1A2 and CYP3A4 activities; DNA adduct formation	[43]
		Allicin	↓CYP2E1 activity (binding to its amino acid residues); *CYP2E1* expression<br>↑hepatic GST and UGT activities; GSTA5 and AFAR levels	[46, 47]
		Taurine	↑serum GSH concentration; GSHS activity (GSH synthesis);	[48]

			↓ GSSG level	
		GSH	↑endogenous intracellular biochemical production	[49]
	Cytoprotective actions	SFN	activate Keap1-Nrf2 signaling pathway (modify Keap1 cysteine residues and/or induce epigenetic modulation of HDACs and DNMTs)	[50]
		Taurine	↓MDA concentration;<br>↑ROS clearance rate; T-AOC; serum activities of SOD, GPx, CAT, and POD; *HO-1*, *GPx1*, *SOD1*, and *SOD2* expression	[48]
		SFN	↓ERK1/2, JNK, p38 MAPK, and NF-κB p65 phosphorylation; *TNF-α*, *IL-1β*, *IL-6*, and *iNOS* expression	[51]
		Taurine	↓*BAX*, *cyt c*, *caspase-9*, *caspase-3*, and *AIFM1* expression;<br>↑*BCL2* expression	[52]
		allicin	↓DNA damage; cell proliferation, angiogenesis, and metastatic processes;<br>↑cell death	[53]
Other promising natural agents	Metabolic intervention	LYC	↓CYP1A1, CYP2A6, and CYP2E1 activities; AFBO, AFP1, AFM1, AFQ1, and AFB1-DNA adducts formation;<br>↑GSH and GSTs levels; AFBO-GSH and AFB1-dialcohol production	[54]
		ASTA	↓NRs (*ahr*, *pxr*, and *car*) and CYP450 (*cyp1a1*, *cyp1a5*, *cyp2c18*, *cyp2d6*, and *cyp3a9*) expression<br>↑GSH levels; *GCLC* and *GPx* expression	[55, 56]
		CUR	↑overall DNA methylation level; *DNMT1*, *DNMT3a*, and *DNMT3b* expression; *GSTA3* and *GSTM2* expression; GSTs enzyme activity<br>↓*CYP1A1*, *CYP1A2*, *CYP3A4*, and *CYP2A6* expression	[57, 58]

	Res	↓the total CYP450 content; *CYP1A1* and *CYP3A4* expression;<br>↑GSH content; GST activity; and *GST*, *GCLC*, and *UGT1A8* expression	[59, 60]
Cytoprotective actions	ASTA	quench singlet oxygen and scavenge free radicals;<br>↑Nrf2 nuclear translocation; *NQO1*, *HO-1*, and *GPx* expression	[56]
	LYC	↑Nrf2 nuclear translocation; *HO-1* and *NQO1* expression; and GPx, SOD, and T-AOC contents	[61]
	CUR	↓phospho-NF-κB p65 expression and nuclear translocation; pro-inflammatory mediators production	[62, 63]
	Res	↓JNK and p38 MAPK signaling pathways; TNF-α, IL-1β, IL-6, and MCP-1 levels; cell proliferation;<br>↑cell apoptosis	[64]
	ASTA	act against cell apoptosis;<br>↓p-ERK/ERK, cyt c, caspase-3, caspase-9, and BAX/BCL-2 ratio	[65]
	LYC	exhibit potential anticancer effects;<br>quench singlet oxygen; simulate cytoprotective enzymes production, initiate apoptosis, inhibit cell proliferation and cell cycle progression	[]

Reference

[1] Wang, L.; Huang, Q.; Wu, J.; Wu, W.; Jiang, J.; Yan, H.; Huang, J.; Sun, Y.; Deng, Y. The metabolism and biotransformation of AFB(1): Key enzymes and pathways. *Biochem. Pharmacol.* **2022**, *199*, 115005. https://doi.org/10.1016/j.bcp.2022.115005.

[2] Deng, J.; Zhao, L.; Zhang, N.Y.; Karrow, N.A.; Krumm, C.S.; Qi, D.S.; Sun, L.H. Aflatoxin B(1) metabolism: Regulation by phase I and II metabolizing enzymes and chemoprotective agents. *Mutat. Res. Rev. Mutat.* **2018**, *778*, 79-89. https://doi.org/10.1016/j.mrrev.2018.10.002.

[3] Santos, A.R.; Carreiro, F.; Freitas, A.; Barros, S.; Brites, C.; Ramos, F.; Sanches Silva, A. Mycotoxins Contamination in Rice: Analytical Methods, Occurrence and Detoxification Strategies. *Toxins (Basel)* **2022**, *14*, 647. https://doi.org/10.3390/toxins14090647.

[4] Wieckowska, M.; Szelenberger, R.; Niemcewicz, M.; Harmata, P.; Poplawski, T.; Bijak, M. Ochratoxin A-The Current Knowledge Concerning Hepatotoxicity, Mode of Action and Possible Prevention. *Molecules* **2023**, *28*, 6617. https://doi.org/10.3390/molecules28186617.

[5] Gu, K.; Ryu, D.; Lee, H.J. Ochratoxin A and its reaction products affected by sugars during heat processing. *Food Chem.* **2021**, *348*, 129038. https://doi.org/10.1016/j.foodchem.2021.129038.

[6] Yu, H.; Zhang, J.; Chen, Y.; Zhu, J. Zearalenone and Its Masked Forms in Cereals and Cereal-Derived Products: A Review of the Characteristics, Incidence, and Fate in Food Processing. *J. Fungi (Basel)* **2022**, *8*, 976. https://doi.org/10.3390/jof8090976.

[7] Han, X.; Huangfu, B.; Xu, T.; Xu, W.; Asakiya, C.; Huang, K.; He, X. Research Progress of Safety of Zearalenone: A Review. *Toxins (Basel)* **2022**, *14*, 386. https://doi.org/10.3390/toxins14060386.

[8] Chen, J.; Lei, X.; Wang, Q.; Liu, H.; Liu, J. Zearalenone degradation by peptide-based enzyme mimics attached membrane reactor: Performance and mechanism. *Food Chem.* **2025**, *463*, 141399. https://doi.org/10.1016/j.foodchem.2024.141399

[9] Yue, J.; Guo, D.; Gao, X.; Wang, J.; Nepovimova, E.; Wu, W.; Kuca, K. Deoxynivalenol (Vomitoxin)-Induced Anorexia Is Induced by the Release of Intestinal Hormones in Mice. *Toxins* **2021**, *13*, 512.

[10] Khodaei, D.; Javanmardi, F.; Khaneghah, A.M. The global overview of the occurrence of mycotoxins in cereals: a three-year survey. *Curr. Opin. Food Sci.* **2021**, *39*, 36-42. https://doi.org/10.1016/j.cofs.2020.12.012.

[11] Chen, J.; Wen, J.; Tang, Y.; Shi, J.; Mu, G.; Yan, R.; Cai, J.; Long, M. Research Progress on Fumonisin B1 Contamination and Toxicity: A Review. *Molecules* **2021**, *26*, 5238. https://doi.org/10.3390/molecules26175238.

[12] Alsulami, T.; Nath, N.; Flemming, R.; Wang, H.; Zhou, W.; Yu, J.H. Development of a novel homogeneous immunoassay using the engineered luminescent enzyme NanoLuc for the quantification of the mycotoxin fumonisin B1. *Biosens Bioelectron* **2021**, *177*, 112939. https://doi.org/10.1016/j.bios.2020.112939.

[13] Twarużek, M.; Ałtyn, I.; Kosicki, R. Dietary Supplements Based on Red Yeast Rice—A Source of Citrinin?. *Toxins* **2021**, *13*, 497. https://doi.org/10.3390/toxins13070497.

[14] Caceres, I.; Khoury, A.A.; Khoury, R.E.; Lorber, S.; Oswald, I.P.; Khoury, A.E.; Atoui, A.; Puel, O.; Bailly, J.D. Aflatoxin Biosynthesis and Genetic Regulation: A Review. *Toxins (Basel)* **2020**, *12*, 150. https://doi.org/10.3390/toxins12030150.

[15] Kolawole, O.; Meneely, J.; Petchkongkaew, A.; Elliott, C. A review of mycotoxin biosynthetic pathways: associated genes and their expressions under the influence of climatic factors. *Fungal Biol. Rev.* **2021**, *37*, 8-26. https://doi.org/10.1016/j.fbr.2021.04.003.
[16] Yu, J.; Bhatnagar, D.; Cleveland, T.E. Completed sequence of aflatoxin pathway gene cluster in Aspergillus parasiticus. *FEBS Lett.* **2004**, *564*, 126-130. https://doi.org/10.1016/S0014-5793(04)00327-8.
[17] Ehrlich, K.C.; Montalbano, B.; Boue, S.M.; Bhatnagar, D. An aflatoxin biosynthesis cluster gene encodes a novel oxidase required for conversion of versicolorin a to sterigmatocystin. *Appl. Environ. Microb.* **2005**, *71*, 8963-8965. https://doi.org/10.1128/AEM.71.12.8963-8965.2005.
[18] Wang, Y.; Wang, L.; Wu, F.; Liu, F.; Wang, Q.; Zhang, X.; Selvaraj, J.N.; Zhao, Y.; Xing, F.; Yin, W.B.; et al. A Consensus Ochratoxin A Biosynthetic Pathway: Insights from the Genome Sequence of Aspergillus ochraceus and a Comparative Genomic Analysis. *Appl. Environ. Microb.* **2018**, *84*, e01009-01018. https://doi.org/10.1128/aem.01009-18.
[19] Ferrara, M.; Gallo, A.; Perrone, G.; Magistà, D.; Baker, S.E. Comparative Genomic Analysis of Ochratoxin A Biosynthetic Cluster in Producing Fungi: New Evidence of a Cyclase Gene Involvement. *Front. Microbiol.* **2020**, *11*, 581309. https://doi.org/10.3389/fmicb.2020.581309.
[20] Stakheev, A.A.; Erokhin, D.V.; Meleshchuk, E.A.; Mikityuk, O.D.; Statsyuk, N.V. Effect of Compactin on the Mycotoxin Production and Expression of Related Biosynthetic and Regulatory Genes in Toxigenic Fusarium culmorum. *Microorganisms* **2022**, *10*, 1347. https://doi.org/10.3390/microorganisms10071347.
[21] Dai, C.; Sharma, G.; Liu, G.; Shen, J.; Shao, B.; Hao, Z. Therapeutic detoxification of quercetin for aflatoxin B1-related toxicity: Roles of oxidative stress, inflammation, and metabolic enzymes. *Environ. Pollut.* **2024**, *345*, 123474. https://doi.org/10.1016/j.envpol.2024.123474.
[22] Owumi, S.E.; Ajakaiye, B.; Akinwunmi, A.O.; Nwozo, S.O.; Oyelere, A.K. The Hydrophobic Extract of Sorghum bicolor (L. Moench) Enriched in Apigenin-Protected Rats against Aflatoxin B1-Associated Hepatorenal Derangement. *Molecules* **2023**, *28*, 3013. https://doi.org/10.3390/molecules28073013.
[23] Wang, X.; Wang, T.; Nepovimova, E.; Long, M.; Wu, W.; Kuca, K. Progress on the detoxification of aflatoxin B1 using natural anti-oxidants. *Food Chem. Toxicol.* **2022**, *169*, 113417. https://doi.org/10.1016/j.fct.2022.113417.
[24] Tsega, S.A.; Manoj, V.R.; Gebretsadik, M.H.; Lumsangkul, C.; Chen, Y.P. Catechin and quercitrin mitigate the cytotoxic effects of aflatoxin-B1 on liver and colon cells by inhibiting cytochrome P450 1A2 and 3A4, in silico. *Food Biosci.* **2025**, *64*, 105989. https://doi.org/10.1016/j.fbio.2025.105989.
[25] Liu, J.; Liu, Y.; Huang, C.; He, C.; Yang, T.; Ren, R.; Xin, Z.; Wang, X. Quercetin-Driven Akkermansia Muciniphila Alleviates Obesity by Modulating Bile Acid Metabolism via an ILA/m(6)A/CYP8B1 Signaling. *Adv Sci (Weinh)* **2025**, *12*, e2412865. https://doi.org/10.1002/advs.202412865.
[26] Erichsen, T.J.; Aehlen, A.; Ehmer, U.; Kalthoff, S.; Manns, M.P.; Strassburg, C.P. Regulation of the human bile acid UDP-glucuronosyltransferase 1A3 by the farnesoid X receptor and bile acids. *J. Hepatol.* **2010**, *52*, 570-578. https://doi.org/10.1016/j.jhep.2010.01.010.

[27] Zhao, Y.; Zhang, X.; Zhang, N.; Zhou, Q.; Fan, D.; Wang, M. Lipophilized apigenin derivatives produced during the frying process as novel antioxidants. *Food Chem.* **2022**, *379*, 132178. https://doi.org/10.1016/j.foodchem.2022.132178.

[28] Lv, J.M.; Gouda, M.; Ye, X.Q.; Shao, Z.P.; Chen, J.C. Evaluation of Proanthocyanidins from Kiwi Leaves (Actinidia chinensis) against Caco-2 Cells Oxidative Stress through Nrf2-ARE Signaling Pathway. *Antioxidants* **2022**, *11*, 1367. https://doi.org/10.3390/antiox11071367.

[29] Li, T.; Li, F.; Liu, X.; Liu, J.; Li, D. Synergistic anti-inflammatory effects of quercetin and catechin via inhibiting activation of TLR4–MyD88-mediated NF-κB and MAPK signaling pathways. *Phytother. Res.* **2019**, *33*, 756-767. https://doi.org/10.1002/ptr.6268.

[30] Dai, C.; Zhang, Q.; Shen, L.; Sharma, G.; Jiang, H.; Wang, Z.; Shen, J. Quercetin Attenuates Quinocetone-Induced Cell Apoptosis In Vitro by Activating the P38/Nrf2/HO-1 Pathway and Inhibiting the ROS/Mitochondrial Apoptotic Pathway. *Antioxidants* **2022**, *11*, 1498. https://doi.org/10.3390/antiox11081498.

[31] Ahmed, S.A.; Parama, D.; Daimari, E.; Girisa, S.; Banik, K.; Harsha, C.; Dutta, U.; Kunnumakkara, A.B. Rationalizing the therapeutic potential of apigenin against cancer. *Life Sci.* **2021**, *267*, 118814. https://doi.org/10.1016/j.lfs.2020.118814.

[32] Wang, X.; He, Y.; Tian, J.; Muhammad, I.; Liu, M.; Wu, C.; Xu, C.; Zhang, X. Ferulic acid prevents aflatoxin B1-induced liver injury in rats via inhibiting cytochrome P450 enzyme, activating Nrf2/GST pathway and regulating mitochondrial pathway. *Ecotoxicol Environ. Saf.* **2021**, *224*, 112624. https://doi.org/10.1016/j.ecoenv.2021.112624.

[33] Wang, X.; Yang, F.; Na, L.; Jia, M.; Ishfaq, M.; Zhang, Y.; Liu, M.; Wu, C. Ferulic acid alleviates AFB1-induced duodenal barrier damage in rats via up-regulating tight junction proteins, down-regulating ROCK, competing CYP450 enzyme and activating GST. *Ecotox. Environ. Safe.* **2022**, *241,* 113805. https://doi.org/10.1016/j.ecoenv.2022.113805.

[34] Liu, Z.; Yang, Y.; Xu, Y.; Zhang, Z.; Tang, R.; Liu, J.; Jiang, H.; Zhao, R. Procyanidin B1 and p-coumaric acid from whole highland barley ameliorated HFD-induced impaired glucose tolerance via small intestinal barrier and hepatic glucose metabolism. *Food Funct.* **2024**, *15*, 9272-9283. https://doi.org/10.1039/d4fo02805h.

[35] Owumi, S.E.; Bello, S.A.; Najophe, S.E.; O. Nwozo, S.; O. Esan, I. Coadministration of gallic acid abates zearalenone-mediated defects in male rat's reproductive function. *J. Biochem. Mol. Toxic.* **2021**, *36*, e22940. https://doi.org/10.1002/jbt.22940.

[36] Cheng, K.; Niu, J.; Zhang, J.; Qiao, Y.; Dong, G.; Guo, R.; Zheng, X.; Song, Z.; Huang, J.; Wang, J.; et al. Hepatoprotective effects of chlorogenic acid on mice exposed to aflatoxin B1: Modulation of oxidative stress and inflammation. *Toxicon* **2023**, *231*, 107177. https://doi.org/10.1016/j.toxicon.2023.107177.

[37] Zhao, X.L.; Yu, L.; Zhang, S.D.; Ping, K.; Ni, H.Y.; Qin, X.Y.; Zhao, C.J.; Wang, W.; Efferth, T.; Fu, Y.J. Cryptochlorogenic acid attenuates LPS-induced inflammatory response and oxidative stress via upregulation of the Nrf2/HO-1 signaling pathway in RAW 264.7 macrophages. *Int. Immunopharmacol.* **2020**, *83*, 106436. https://doi.org/10.1016/j.intimp.2020.106436.

[38] Luo, J.; Wu, X.; Chen, D.; Yu, B.; He, J. Dietary ferulic acid supplementation enhances antioxidant capacity and alleviates hepatocyte pyroptosis in diquat challenged piglets. *J. Anim. Sci. Biotechnol.* **2024**, *15*, 134. https://doi.org/10.1186/s40104-024-01086-5.
[39] Kose, T.; Sharp, P.A.; Latunde-Dada, G.O. Upregulation of Nrf2 Signalling and the Inhibition of Erastin-Induced Ferroptosis by Ferulic Acid in MIN6 Cells. *Int. J. of Mol. Sci.* **2022**, *23*, 15886. https://doi.org/10.3390/ijms232415886.
[40] Li, D.; Wan, M.; Xue, L.; Zhang, Z.; Qiu, Y.; Mei, F.; Tang, N.; Yu, C.; Yu, Y.; Chen, T.; et al. Zinc promotes microbial p-coumaric acid production that protects against cholestatic liver injury. *Cell Host & Microbe* **2024**, *32*, 2195-2211.e9. https://doi.org/10.1016/j.chom.2024.11.002.
[41] Bai, J.; Zhang, Y.; Tang, C.; Hou, Y.; Ai, X.; Chen, X.; Zhang, Y.; Wang, X.; Meng, X. Gallic acid: Pharmacological activities and molecular mechanisms involved in inflammation-related diseases. *Biomed. Pharmacother.* **2021**, *133*, 110985. https://doi.org/10.1016/j.biopha.2020.110985.
[42] Gupta, A.; Atanasov, A.G.; Li, Y.; Kumar, N.; Bishayee, A. Chlorogenic acid for cancer prevention and therapy: Current status on efficacy and mechanisms of action. *Pharmacol. Res.* **2022**, *186*, 106505. https://doi.org/10.1016/j.phrs.2022.106505.
[43] Gross-Steinmeyer, K.; Stapleton, P.L.; Tracy, J.H.; Bammler, T.K.; Strom, S.C.; Eaton, D.L. Sulforaphane- and Phenethyl Isothiocyanate–Induced Inhibition of Aflatoxin B1–Mediated Genotoxicity in Human Hepatocytes: Role of GSTM1 Genotype and CYP3A4 Gene Expression. *Toxicol. Sci.* **2010**, *116*, 422-432. https://doi.org/10.1093/toxsci/kfq135.
[44] Fiala, J.L.A.; Egner, P.A.; Wiriyachan, N.; Ruchirawat, M.; Kensler, K.H.; Wogan, G.N.; Groopman, J.D.; Croy, R.G.; Essigmann, J.M. Sulforaphane-Mediated Reduction of Aflatoxin B1-N7-Guanine in Rat Liver DNA: Impacts of Strain and Sex. *Toxicol. Sci.* **2011**, *121*, 57-62. https://doi.org/10.1093/toxsci/kfr026.
[45] He, C.; Gao, M.; Zhang, X.; Lei, P.; Yang, H.; Qing, Y.; Zhang, L. The Protective Effect of Sulforaphane on Dextran Sulfate Sodium-Induced Colitis Depends on Gut Microbial and Nrf2-Related Mechanism. *Front. Nutr.* **2022**, *9*, 893344. https://doi.org/10.3389/fnut.2022.893344.
[46] Nan, B.; Yang, C.; Li, L.; Ye, H.; Yan, H.; Wang, M.; Yuan, Y. Allicin alleviated acrylamide-induced NLRP3 inflammasome activation via oxidative stress and endoplasmic reticulum stress in Kupffer cells and SD rats liver. *Food Chem. Toxicol.* **2021**, *148*, 111937. https://doi.org/10.1016/j.fct.2020.111937.
[47] Berges, R. Comparison of the chemopreventive efficacies of garlic powders with different alliin contents against aflatoxin B1 carcinogenicity in rats. *Carcinogenesis* **2004**, *25*, 1953-1959. https://doi.org/10.1093/carcin/bgh200.
[48] Wang, L.; Jiang, L.; Chu, Y.; Feng, F.; Tang, W.; Chen, C.; Qiu, Y.; Hu, Z.; Diao, H.; Tang, Z. Dietary Taurine Improves Growth Performance and Intestine Health via the GSH/GSSG Antioxidant System and Nrf2/ARE Signaling Pathway in Weaned Piglets. *Antioxidants* **2023**, *12*, 1852. https://doi.org/10.3390/antiox12101852.
[49] Al-Temimi, A.A.; Al-Mossawi, A.E.B.; Al-Hilifi, S.A.; Korma, S.A.; Esatbeyoglu, T.; Rocha, J.M.; Agarwal, V. Glutathione for Food and Health Applications with Emphasis on Extraction, Identification, and Quantification Methods: A Review. *Metabolites* **2023**, *13*, 465. https://doi.org/10.3390/metabo13040465.

[50] Kaiser, A.E.; Baniasadi, M.; Giansiracusa, D.; Giansiracusa, M.; Garcia, M.; Fryda, Z.; Wong, T.L.; Bishayee, A. Sulforaphane: A Broccoli Bioactive Phytocompound with Cancer Preventive Potential. *Cancers* **2021**, *13*, 4796. https://doi.org/10.3390/cancers13194796.
[51] Qin, S.; Yang, C.; Huang, W.; Du, S.; Mai, H.; Xiao, J.; Lü, T. Sulforaphane attenuates microglia-mediated neuronal necroptosis through down-regulation of MAPK/NF-κB signaling pathways in LPS-activated BV-2 microglia. *Pharmacol. Res.* **2018**, *133*, 218-235. https://doi.org/10.1016/j.phrs.2018.01.014.
[52] Ji, X.; Ding, H.; Zhou, F.; Zhang, F.; Wu, D. Taurine ameliorates deoxynivalenol-induced intestinal injury in piglets: Restoration of mitochondrial function linked to the PGC1α-NRF1/2 axis. *Ecotox. Environ. Safe.* **2025**, *292*, 117938. https://doi.org/10.1016/j.ecoenv.2025.117938.
[53] Catanzaro, E.; Canistro, D.; Pellicioni, V.; Vivarelli, F.; Fimognari, C. Anticancer potential of allicin: A review. *Pharmacol. Res.* **2022**, *177*, 106118. https://doi.org/10.1016/j.phrs.2022.106118.
[54] Li, M.; Tang, S.; Peng, X.; Sharma, G.; Yin, S.; Hao, Z.; Li, J.; Shen, J.; Dai, C. Lycopene as a Therapeutic Agent against Aflatoxin B1-Related Toxicity: Mechanistic Insights and Future Directions. *Antioxidants* **2024**, *13*, 452. https://doi.org/10.3390/antiox13040452.
[55] Zou, Y.; Zhang, S.; Yang, J.; Qin, C.; Jin, B.; Liang, Z.; Yang, S.; Li, L.; Long, M. Protective Effects of Astaxanthin on Ochratoxin A-Induced Liver Injury: Effects of Endoplasmic Reticulum Stress and Mitochondrial Fission-Fusion Balance. *Toxins (Basel)* **2024**, *16*, 68. https://doi.org/10.3390/toxins16020068.
[56] Li, L.; Chen, Y.; Jiao, D.; Yang, S.; Li, L.; Li, P. Protective Effect of Astaxanthin on Ochratoxin A-Induced Kidney Injury to Mice by Regulating Oxidative Stress-Related NRF2/KEAP1 Pathway. *Molecules* **2020**, *25*, 1386. https://doi.org/10.3390/molecules25061386.
[57] Liu, R.; Ding, Y.; Li, W.; Li, S.; Li, X.; Zhao, D.; Zhang, Y.; Wei, G.; Zhang, X. Protective role of curcumin on broiler liver by modulating aflatoxin B1-induced DNA methylation and CYPs expression. *Ecotox. Environ. Safe.* **2023**, *260*, 115086. https://doi.org/10.1016/j.ecoenv.2023.115086.
[58] Muhammad, I.; Wang, H.; Sun, X.; Wang, X.; Han, M.; Lu, Z.; Cheng, P.; Hussain, M.A.; Zhang, X. Dual Role of Dietary Curcumin Through Attenuating AFB1-Induced Oxidative Stress and Liver Injury via Modulating Liver Phase-I and Phase-II Enzymes Involved in AFB1 Bioactivation and Detoxification. *Front. Pharmacol.* **2018**, *9*, 554. https://doi.org/10.3389/fphar.2018.00554.
[59] Liu, F.; Wang, Y.; Zhou, X.; Liu, M.; Jin, S.; Shan, A.; Feng, X. Resveratrol Relieved Acute Liver Damage in Ducks (Anas platyrhynchos) Induced by AFB1 via Modulation of Apoptosis and Nrf2 Signaling Pathways. *Animals* **2021**, *11*, 3516. https://doi.org/10.3390/ani11123516.
[60] Zhou, X.; Zhao, Y.; Wang, J.; Wang, X.; Chen, C.; Yin, D.; Zhao, F.; Yin, J.; Guo, M.; Zhang, L.; et al. Resveratrol represses estrogen-induced mammary carcinogenesis through NRF2-UGT1A8-estrogen metabolic axis activation. *Biochem. Pharmacol.* **2018**, *155*, 252-263. https://doi.org/10.1016/j.bcp.2018.07.006.
[61] Wang, Y.; Liu, Z.; Ma, J.; Xv, Q.; Gao, H.; Yin, H.; Yan, G.; Jiang, X.; Yu, W. Lycopene attenuates the inflammation and apoptosis in aristolochic acid nephropathy by targeting the

Nrf2 antioxidant system. *Redox Biol.* **2022**, *57*, 102494. https://doi.org/10.1016/j.redox.2022.102494.
[62] Liu, C.; Yan, X.; Zhang, Y.; Yang, M.; Ma, Y.; Zhang, Y.; Xu, Q.; Tu, K.; Zhang, M. Oral administration of turmeric-derived exosome-like nanovesicles with anti-inflammatory and pro-resolving bioactions for murine colitis therapy. *J. Nanobiotechnol.* **2022**, *20*, 206. https://doi.org/10.1186/s12951-022-01421-w.
[63] Zhao, J.; Jia, W.; Zhang, R.; Wang, X.; Zhang, L. Improving curcumin bioavailability: Targeted delivery of curcumin and loading systems in intestinal inflammation. *Food Res. Int.* **2024**, *196*, 115079. https://doi.org/10.1016/j.foodres.2024.115079.
[64] Yang, G.; Chang, C.C.; Yang, Y.; Yuan, L.; Xu, L.; Ho, C.T.; Li, S. Resveratrol Alleviates Rheumatoid Arthritis via Reducing ROS and Inflammation, Inhibiting MAPK Signaling Pathways, and Suppressing Angiogenesis. *J. Agric. Food Chem.* **2018**, *66*, 12953-12960. https://doi.org/10.1021/acs.jafc.8b05047.
[65] Fakhri, S.; Yosifova Aneva, I.; Farzaei, M.H.; Sobarzo-Sánchez, E. The Neuroprotective Effects of Astaxanthin: Therapeutic Targets and Clinical Perspective. *Molecules* **2019**, *24*, 2640. https://doi.org/10.3390/molecules24142640.
[66] Ozkan, G.; Günal-Köroğlu, D.; Karadag, A.; Capanoglu, E.; Cardoso, S.M.; Al-Omari, B.; Calina, D.; Sharifi-Rad, J.; Cho, W.C. A mechanistic updated overview on lycopene as potential anticancer agent. *Biomed. Pharmacother.* **2023**, *161*, 114428. https://doi.org/10.1016/j.biopha.2023.114428.
